# Immune checkpoints are predominantly co-expressed by clonally expanded CD4^+^FoxP3^+^ intratumoral T-cells in primary human cancers

**DOI:** 10.1186/s13046-023-02897-6

**Published:** 2023-12-06

**Authors:** Delphine Bredel, Edi Tihic, Séverine Mouraud, François-Xavier Danlos, Sandrine Susini, Marine Aglave, Alexia Alfaro, Chifaou Mohamed-Djalim, Mathieu Rouanne, Héloise Halse, Amélie Bigorgne, Lambros Tselikas, Stéphane Dalle, Dana M. Hartl, Eric Baudin, Catherine Guettier, Eric Vibert, Olivier Rosmorduc, Caroline Robert, Sophie Ferlicot, Bastien Parier, Laurence Albiges, Vincent Thomas de Montpreville, Benjamin Besse, Olaf Mercier, Caroline Even, Ingrid Breuskin, Marion Classe, Camélia Radulescu, Thierry Lebret, Patricia Pautier, Sébastien Gouy, Jean-Yves Scoazec, Laurence Zitvogel, Aurélien Marabelle, Mélodie Bonvalet

**Affiliations:** 1 Gustave Roussy, 114 Rue Édouard Vaillant, 94805 Villejuif, France; 2 Institut National de La Santé Et de La Recherche Médicale (INSERM) U1015, Laboratoire de Recherche Translationnelle en Immunothérapie (LRTI), 94805 Villejuif, France; 3 Institut National de La Santé Et de La Recherche Médicale (INSERM) CIC1428, Centre d’Investigation Clinique BIOTHERIS, 94805 Villejuif, France; 4 Université Paris-Saclay, Faculté de Médecine, 94270 Le Kremlin-Bicêtre, France; 5 Gustave Roussy, Département d’Innovation Thérapeutique Et d’Essais Précoces (DITEP), 94805 Villejuif, France; 6 Gustave Roussy, Plateforme de bioinformatique, F-94805 Villejuif, France; 7 Gustave Roussy, Université Paris-Saclay, UMS 23/3655, Plateforme Imagerie Et Cytométrie, Villejuif, France; 8 Department of Microbiology and Immunology, Vagelos College of Physicians and Surgeons, Columbia University, New York, USA; 9 Institut National de La Santé Et de La Recherche Médicale (INSERM) U1163, Institut Imagine, Université Paris Descartes, 75015 Paris, France; 10 Gustave Roussy, Université Paris Saclay, Département d’Anesthésie, Chirurgie et Imagerie Interventionnelle, F-94805 Villejuif, France; 11 Department of Dermatology, HCL Cancer Institute, Lyon Cancer Research Center, 69495 Lyon, France; 12 Gustave Roussy, Département d’Oncologie Médicale, F-94805 Villejuif, France; 13 Service d’Anatomie Pathologique, Hôpital Bicêtre, AP-HP, 94270 Le Kremlin-Bicêtre, France; 14 UMR-S 1193, Hôpital Paul Brousse Université Paris Saclay, 94800 Villejuif, France; 15 Centre Hépato-Biliaire, Hôpital Paul Brousse, AP-HP, 94800 Villejuif, France; 16 Institut National de La Santé Et de La Recherche Médicale (INSERM) U981, Gustave Roussy, 94805 Villejuif, France; 17 Centre National de Recherche Scientifique (CNRS), Gustave Roussy, Université Paris-Saclay, UMR 9019, 94805 Villejuif, France; 18 Service de Chirurgie Urologique, Hôpital Bicêtre, AP-HP, Le Kremlin-Bicêtre, France; 19 Département de Pathologie, Hôpital Marie-Lannelongue, Le Plessis-Robinson, France; 20 Service de Chirurgie Thoracique Et Transplantation Cardio-Pulmonaire, Hôpital Marie-Lannelongue, UMR_S 999 INSERM, Université Paris-Saclay, GHPSJ, 92350 Le Plessis-Robinson, France; 21 Gustave Roussy, Département de Biopathologie, F-94805 Villejuif, France; 22 Département de Pathologie, Hôpital Foch, UVSQ, Université Paris-Saclay, 92150 Suresnes, France; 23 Département d’Urologie, Hôpital Foch, UVSQ-Université Paris-Saclay, 92150 Suresnes, France

**Keywords:** Cancer, Immunology, Immune checkpoints, T-cells, Immunotherapy, Single-cell RNA-Seq, Flow cytometry, TCR repertoire

## Abstract

**Background:**

In addition to anti-PD(L)1, anti-CTLA-4 and anti-LAG-3, novel immune checkpoint proteins (ICP)-targeted antibodies have recently failed to demonstrate significant efficacy in clinical trials. In these trials, patients were enrolled without screening for drug target expression. Although these novel ICP-targeted antibodies were expected to stimulate anti-tumor CD8 + T-cells, the rationale for their target expression in human tumors relied on pre-clinical IHC stainings and transcriptomic data, which are poorly sensitive and specific techniques for assessing membrane protein expression on immune cell subsets. Our aim was to describe ICP expression on intratumoral T-cells from primary solid tumors to better design upcoming neoadjuvant cancer immunotherapy trials.

**Methods:**

We prospectively performed multiparameter flow cytometry and single-cell RNA sequencing (scRNA-Seq) paired with TCR sequencing on freshly resected human primary tumors of various histological types to precisely determine ICP expression levels within T-cell subsets.

**Results:**

Within a given tumor type, we found high inter-individual variability for tumor infiltrating CD45 + cells and for T-cells subsets. The proportions of CD8^+^ T-cells (~ 40%), CD4^+^ FoxP3^-^ T-cells (~ 40%) and CD4^+^ FoxP3^+^ T-cells (~ 10%) were consistent across patients and indications. Intriguingly, both stimulatory (CD25, CD28, 4-1BB, ICOS, OX40) and inhibitory (PD-1, CTLA-4, PD-L1, CD39 and TIGIT) checkpoint proteins were predominantly co-expressed by intratumoral CD4^+^FoxP3^+^ T-cells. ScRNA-Seq paired with TCR sequencing revealed that T-cells with high clonality and high ICP expressions comprised over 80% of FoxP3^+^ cells among CD4^+^ T-cells. Unsupervised clustering of flow cytometry and scRNAseq data identified subsets of CD8^+^ T-cells and of CD4^+^ FoxP3^-^ T-cells expressing certain checkpoints, though these expressions were generally lower than in CD4^+^ FoxP3^+^ T-cell subsets, both in terms of proportions among total T-cells and ICP expression levels.

**Conclusions:**

Tumor histology alone does not reveal the complete picture of the tumor immune contexture. In clinical trials, assumptions regarding target expression should rely on more sensitive and specific techniques than conventional IHC or transcriptomics. Flow cytometry and scRNAseq accurately characterize ICP expression within immune cell subsets. Much like in hematology, flow cytometry can better describe the immune contexture of solid tumors, offering the opportunity to guide patient treatment according to drug target expression rather than tumor histological type.

**Supplementary Information:**

The online version contains supplementary material available at 10.1186/s13046-023-02897-6.

## Introduction

Cancer development often entails an excess of host immune tolerance generated by regulatory T-cells, also known as "Tregs" (typically CD4 + FoxP3 + T-cells), over effector CD8 + and CD4 + Foxp3- anti-tumor T-cells in the tumor microenvironment [1]. Both types of T-cells recognize tumor-specific epitopes and neo-antigens presented to their T-cell receptor (TCR) by MHC class-I and -II molecules. T-cell activation and proliferation depend on the integration of co-stimulatory and co-inhibitory signals provided by immune checkpoint (ICP) proteins at the synapse between T-cells and antigen-presenting cells [2]. Antagonistic monoclonal antibodies (mAbs) directed against the co-inhibitory checkpoint molecules PD-1, PD-L1, CTLA-4 and LAG-3 have revolutionized the treatment of multiple cancers and established immunotherapy as a new standard of care in oncology over the past 13 years.

Many other ICP targeted-antibodies have recently been developed, designed to be antagonistic against other T-cell co-inhibitory ICPs (e.g., TIGIT, CD39) [3, 4] or to be agonistic against co-stimulatory ICPs (4-1BB, ICOS, OX40) [5–18]. However, most of these new immunotherapies have failed or are facing challenges in ongoing oncology clinical trials due to a lack of clinical activity. Although selected for their ability to stimulate anti-tumor T-cells, the rationale for their target expression in human tumors relied on murine data, human bulk transcriptomic, and single immuno-histo-chemical (IHC) stainings of formalin-fixed tumors, which have limited sensitivity and specificity to identify immune cell subsets (see references in Supplementary Data 1).

Here, we report the protein expression level of ICPs on tumor infiltrating T-cells across various human primary solid tumors. We prospectively collected and analyzed freshly resected human tumors of different types and conducted flow cytometry and single-cell RNA sequencing along with TCR sequencing analyses on CD8 + , CD4 + FoxP3-, and CD4 + FoxP3 + T-cells to study the co-expression of stimulatory and inhibitory ICPs including CD25, CD28, CD39, 4-1BB, CTLA-4, ICOS, OX40, PD-1, PD-L1, and TIGIT. Our findings describe the T-cell subsets and their relative ICP expressions in a large cohort of human primary tumors. Notably, our data sheds light on CD4 + FoxP3 + T-cells. Their comprehensive characterization across a diverse range of human tumors amenable to surgical intervention may be beneficial for informing and enhancing upcoming neoadjuvant clinical trials focused on immune checkpoint targeting.

## Materials and methods

### Patients and cohorts characteristics

Patients over 18 years of age from Gustave Roussy Cancer Campus, Marie Lannelongue, Foch, Paul Brousse and Kremlin-Bicêtre hospitals suffering from histologically confirmed and resectable Non-Small Cell Lung Cancer (NSCLC, *n* = 9), Renal Cell Carcinoma (RCC, *n* = 12), Head & Neck Squamous Cell Carcinoma (HNSCC, *n* = 11), Epithelial Ovarian Cancer (EOC, *n* = 22), Urothelial Carcinoma (UC, *n* = 12), HepatoCarcinoma (HCC, *n* = 2), Metastatic Melanoma (MM, *n* = 7), Thyroid Carcinoma (Thyr, *n* = 1) and neuroendocrine Tumor (NET, *n* = 1) were informed according to the ethical guidelines. Sample and clinical data collection were approved by the appropriate authorities and ethics committee (AC-2013–188; DC-2019–3601; ID-RCB: 2016-A00732-49 & 2008-A00373-52) and conducted between April 2013 and December 2021.

### Tumor infiltrated lymphocyte preparations

Tumor specimens were dissociated as previously described [19]. Tumor samples were digested for one hour using a gentle MACS OctoDissociator (Miltenyi Biotec) in dissociation medium RPMI 1640 (GIBCO, 31,870–025), Collagenase IV at 50 IU/mL (Sigma-Aldrich, C2139), Hyaluronidase at 280 IU/mL (Sigma-Aldrich, H6254), and DNAse I at 30 IU/mL (Sigma-Aldrich, 260913)). Digested samples were diluted in phosphate-buffered saline (PBS at 1X ± EDTA and BSA), filtered through a cell strainer (70 µM) and centrifuged for 10 min at 1800 rpm. All samples were freshly analyzed, except metastatic melanoma lymph node samples that were stored in liquid nitrogen prior phenotypic analyses. This dissociation procedure did not impact the expression of ICP assessed in further analyses (Supplementary Data 2).

For flow cytometric analyses, cell pellets were resuspended in PBS for fluorochrome-coupled mAbs staining. For single-cell RNA sequencing (scRNA-Seq), live CD45 positive cells were sorted using a cell sorter Aria Fusion (BD Biosciences). Cells were stained in PBS containing 0.04% bovine serum albumin (BSA, Sigma-Aldrich, A7030-100G) for 20 min at 4 °C with anti-CD45-FITC mAbs (Biolegend, 304038), washed, and resuspended in PBS 0,04% BSA. 7-AAD (Biolegend, 420404) was added to the cells prior to sorting. At least 50,000 live CD45 positive cells were sorted in PBS 10% BSA, centrifuged for 5 min at 1500 rpm, and resuspended at 2000 cell/µl in PBS 0,04% BSA containing 1X Superase (Sigma-Aldrich, 3335399001). Cell viability was tested using Trypan blue, and samples analyzed displayed 85–91% live cells.

### Flow cytometric analyses

For surface labeling, cells were stained with fluorochrome-coupled mAbs. Anti-CTLA-4-PE (BD Biosciences, 555853) was stained at + 37 °C for 30 min before others surface antibodies were added and incubated at + 4 °C for 20 min and washed. Anti-CD3-BUV395 (BD Biosciences, 563546), anti-CD4-BUV496 (BD Biosciences, 564651), anti-ICOS-BUV737 (BD Biosciences, 749665), anti-CD45-BV805 (BD Biosciences, 564914), anti-CD28-BV421 (BD Biolegend 302930), anti-CD39-BV605 (BD Biosciences, 742522), anti-OX40-BV650 (BD Biosciences, 563658), anti-4-1BB-BV711 (BD Biosciences, 740798), anti-PD-1-BV786 (BD Biosciences, 563789), anti-PD-L1-PE/Dazzle594 (Biolegend, 329732), anti-CD25-PE-Cy7 (Beckman Coulter, A52882), anti-CD8-APC-H7 (BD Biosciences,560179), and anti-TIGIT-APC (Biolegend, 372706) mAbs were used for surface labeling. Zombie aqua fixable viability kit (Biolegend, 423102) was used to exclude dead cells. Cells were then permeabilized with FoxP3/Transcription Factor Staining Buffer Set (Thermo Fischer Scientific, 00–5523-00) prior intracellular staining with anti-FoxP3-FITC (eBiosciences, 11–4776-42) mAbs, according to the manufacturer’s protocol. Cells were acquired on a BD LSR Fortessa TM X-20 flow cytometer (BD Biosciences) with single-stained antibody-capturing beads used for compensation (CompBeads, BD Biosciences, 552843). Data were analyzed with Kaluza Analysis software v2.1 (Beckman Coulter).

Non-supervised analyses were performed using PhenoGraph (Rphenograph version 0.99.1) [20]. FCS files were exported using FlowJO software. Marker expression values were transformed using the auto-logicle transformation function. PhenoGraph clustering was performed using 12 markers and a number of nearest neighbors of 30. UMAP was run with nearest neighbors of 30 and a min_distance of 0.1.

### Single-cell RNA sequencing (scRNA-Seq)

Sample preparation was performed at room temperature. Single-cell suspensions were loaded onto a Chromium Single Cell Chip (10X Genomics) following the manufacturer’s instructions for co-encapsulation with barcoded Gel Beads at a target capture rate of ~ 10,000 cells per sample. Captured mRNAs were barcoded during cDNA synthesis according to the manufacturer’s instructions, using the Chromium Single Cell 5' Library & Gel Bead Kit v1 (10X Genomics) for NSCLC_1, NSCLC_2, and HNSCC_2 samples, or the Chromium Next GEM Single Cell 5' GEM, Library Kit v2 (10X Genomics) for EOC_19 and HCC_1 samples. Samples were processed simultaneously for generating GE libraries (PN-1000020) and GE libraries (PN-1000190) for the NSCLC_1, NSCLC_2, HNSCC_2 samples, and the EOC_19, HCC_1 samples, respectively, as well as TCR libraries (10X Genomics). Libraries from NSCLC_1, NSCLC_2, and HNSCC_2 samples were pooled for sequencing in a single SP Illumina flow cell, and were sequenced with an 8-base index read, a 26-base Read1 containing cell-identifying barcodes and unique molecular identifiers (UMIs), and a 93-base Read2 containing transcript sequences on an Illumina NovaSeq 6000. Libraries from EOC_19 and HCC_1 samples were also pooled for sequencing in a single SP Illumina flow cell, and were sequenced with a 10-base index read (dual index), a 26-base Read1 containing cell-identifying barcodes and unique molecular identifiers (UMIs), and a 90-base Read2 containing transcript sequences on an Illumina NovaSeq 6000.

### ScRNA-Seq data alignment and quality control

BCL-files were converted to Fastq format using bcl2fastq (version 2.20.0.422 from Illumina). Fastqc and fastq-screen were used for quality control and evaluation of assignment to the expected genome species. The Ensembl reference transcriptome v99 for homo sapiens GRCh38 build was used to pseudo-map reads with kallisto using the 'bus' subcommand and parameters for 10X Chromium 5' scRNA-Seq v2 chemistry [21]. The index was made with the kb-python wrapper of kallisto [22, 23]. Barcode correction using whitelist provided by the manufacturer (10X Genomics) and gene-based reads quantification was performed with BUStools [24]. Cell barcode by symbol count table were loaded in R using the BUSpaRse package [25]. To call real cells from empty droplets, the emptyDrops() function from the dropletUtils package was used [26, 27]. Barcodes with *p*-value < 0.001 (Benjamini-Hochberg-corrected) were considered legitimate cells.

The count matrix was filtered to exclude genes detected in less than 5 cells, cells with less than 1000 UMIs or less than 200 detected genes, and cells with mitochondrial transcripts proportion higher than 20%.

Cell cycle scoring was performed using the CellcycleScoring() function from the Seurat package and the cyclone() function from Scran [28, 29]. Doublet cells were identified and discarded using the union of two methods: scDblFinder using default parameters, and scds with its hybrid method using default parameters [30, 31]. It was manually verified that the cells identified as doublets did not correspond to cells in G2M phase.

### Single-cell TCR sequencing analysis

Raw BCL-files were demultiplexed and converted to Fastq format using bcl2fastq (version 2.20.0.422 from Illumina). Reads quality control was performed using fastqc and assignment to the expected genome species evaluated with fastq-screen (version 0.14.0). CellRanger (version 3.1.0 from 10X Genomics) was used to generate single-cell V(D)J sequences and annotations. The annotation was merged with corresponding cell barcode of gene expression. The scRepertoire package was used to process annotation to assign clonotype based on TCR chains.

### Dimensionality reduction and clustering analysis

#### Individual analysis

Seurat was used for data processing. 3000 highly variable genes (HVG) were identified using the FindVariableFeatures() method from Seurat applied on data transformed by its LogNormalize method. The scbfa dimension reduction method was applied on the HVG subset as recommended by the authors [28]. Some per-cell bias factors could be regressed out during the scbfa dimension reduction (depending on the samples). The number of scbfa dimensions to keep for further analysis was evaluated by assessing a range of reduced scbfa spaces using 3 to 49 dimensions, with a step of 2. For each generated scbfa space, Louvain clustering of cells was performed using a range of values for the resolution parameter from 0.1 to 1.2 with a step of 0.1. The optimal space was manually evaluated as the one combination of kept dimensions and clustering resolution resolving the best structure (clusters homogeneity and compacity) in an UMAP. Additionaly, we used the clustree method to assess if the selected optimal space corresponded to a relatively stable position in the clustering results tested for these dimensions/resolution combinations [32].

#### Integration analysis

The scbfa dimension reduction method was used to integrate datasets. The datasets were merged using the merge() function from Seurat, and 3000 HVG were identified similar to the individual analysis. Per-cell bias factors, including the G2M cell cycle score and proportion of mitochondrial transcripts, were regressed out during the scbfa dimension reduction. Batch effect was regressed with other potential biases. The optimal space was manually evaluated using UMAP and a range of combinations of dimensions and clustering resolution (i.e. dimension 27, resolution 1.2). The integration results were compared with individual results of each sample to ensure the batch effect correction did not hide any relevant cellular effects or create side effects.

Cerebro, a cell report browser, was used to visualize UMAP of gene expression interactively [33].

### ScRNA-Seq differential expression analysis

Differential expression analyses were conducted between cluster groups using the Wilcoxon test via the FindMarkers() function in Seurat. Only genes with a minimum log2 fold-change of 0.58 in at least 25% of cells from one group were considered, with FDR-adjusted *p*-values < 0.05 (Benjaminin-Hochberg method).

### General statistical analysis

Data representations and statistical analyses were performed using Excel 2016 (Microsoft Office), Prism 9 (GraphPad San Diego, CA, USA), or R v4.1.3 with various packages including dplyr, EnhancedVolcano, ggplot2, ggpubr, ggrepel, htmlwidgets, networkD3, scales, Seurat, and tidyverse. Box plots display numerical data through quartiles, with each dot representing a tumor sample.

## Results

### Tumor-infiltrating T-cell subsets show a similar distribution across various cancer histological types

**Fig. 1:**
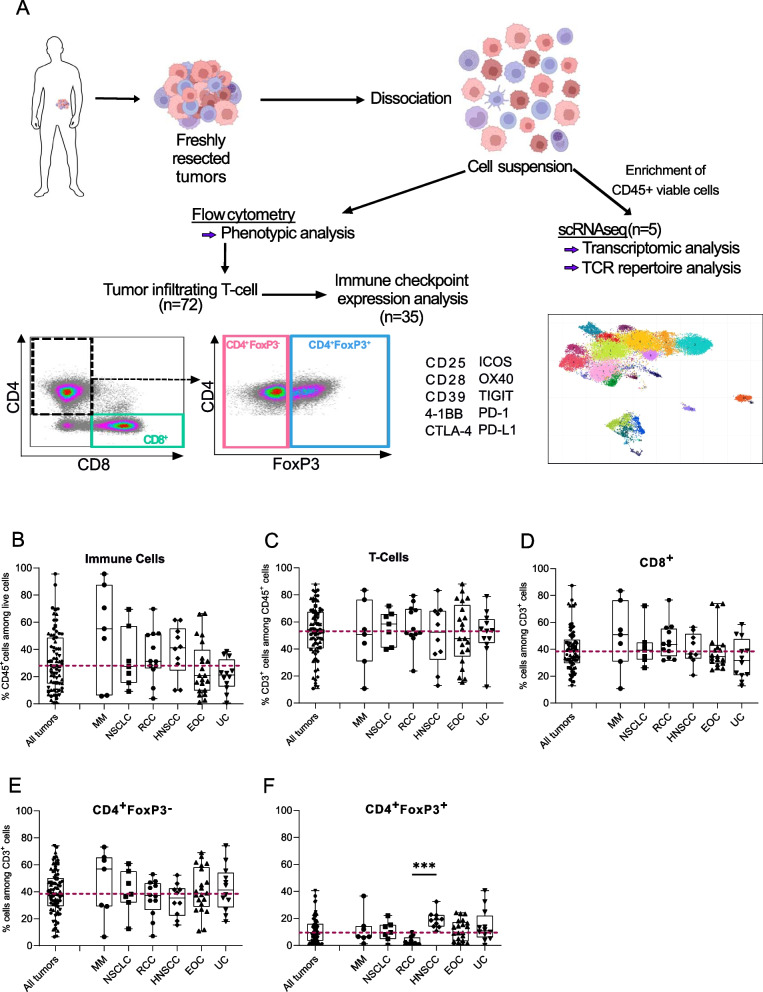
Proportions of T-cell subsets in the tumor microenvironment are independent of tumor histological types. **A** Freshly resected tumors from various histologies (*n* = 72) were collected and dissociated into a cell suspension and stained for T cell subset identification. Immune checkpoints (ICPs) expression was assessed in CD8^+^, CD4^+^FoxP3^−^ and CD4^+^FoxP3^+^ T cells at the protein level using flow cytometry (*n* = 35) and at the transcriptomic level using single-cell RNA sequencing, including TCR sequencing (*n* = 5). Created with BioRender.com. Flow cytometry analysis from 72 fresh tumor specimens. **B** Percentage of CD45^+^ among live cells in the different histologies. **C** Percentage of CD3^+^ T-cells among CD45^+^ cells according to the different histologies. Percentage of CD8^+^ (**D**), CD4^+^FoxP3^−^ (**E**) and CD4^+^FoxP3^+^ (**F**) among CD3^+^ cells in the different histologies. The red dotted line delineates the median of the whole cohort. Dunn’s multiple comparison test, *p value ≤ 0.05; **p value ≤ 0.01; ***p value ≤ 0.001; ****p value ≤ 0.0001. MM: Metastatic Melanoma; NSCLC: Non-Small Cell Lung Carcinoma; RCC: Renal Cell Carcinoma; HNSCC: Head and Neck Squamous Cell Carcinoma; EOC: Epithelial Ovarian Cancer; UC: Urothelial Carcinoma

**Fig. 2:**
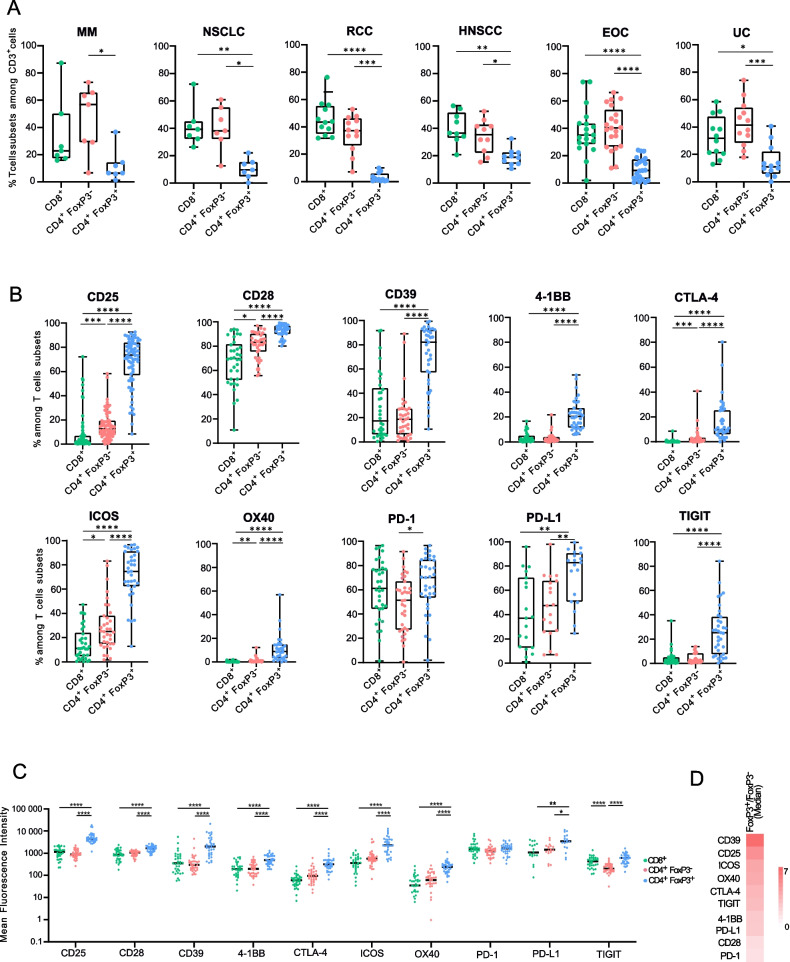
Intratumoral CD4^+^FoxP3^+^ cells make up a small subset but display the highest levels of immune checkpoint protein expression. **A** Proportions of T cell subsets, i.e., CD8^+^, CD4^+^FoxP3^−^ and CD4^+^FoxP3^+^ in MM, NSCLC, RCC, HNSCC, EOC, and UC obtained by flow cytometry analysis of 72 freshly resected tumor specimens. **B** Percentage of immune checkpoint protein (ICP) positive cells in CD8^+^, CD4^+^FoxP3^−^ and CD4 + FoxP3 + T cells from 35 tumor specimens. **C** Mean fluorescence intensity of ICPs in CD8^+^, CD4^+^FoxP3^−^ and CD4^+^FoxP3^+^ T cells from 35 tumor specimens. Dunn’s multiple comparison test was performed independently for each ICP. **D** Heat map displaying the ratio of the ICP median MFI of CD4^+^FoxP3^+^ cells over CD4^+^FoxP3.^−^. Dunn’s multiple comparison test, *p value ≤ 0.05; **p value ≤ 0.01; ***p value ≤ 0.001; ****p value ≤ 0.0001. MM: Metastatic Melanoma; NSCLC: Non-Small Cell Lung Carcinoma; RCC: Renal Cell Carcinoma; HNSCC: Head and Neck Squamous Cell Carcinoma; EOC: Epithelial Ovarian Cancer; UC: Urothelial Carcinoma; ICPs: Immune Checkpoints: (CD25, CD28, CD39, 4-1BB, CTLA-4, ICOS, OX40, PD-1, PD-L1, and TIGIT)

We conducted flow cytometry phenotyping of T-cells in freshly resected human tumors from 72 cancer patients eligible for surgery (Fig. 1A). The cohort included a range of cancer types, including Metastatic Melanoma (MM; *n* = 7), Non-Small Cell Lung Carcinoma (NSCLC; *n* = 7), Renal Cell Carcinoma (RCC; *n* = 12), Head and Neck Squamous Cell Carcinoma (HNSCC; *n* = 10), Epithelial Ovarian Carcinoma (EOC; *n* = 21), Urothelial Carcinoma (UC; *n* = 12), Hepato-Cellular Carcinoma (HCC; *n* = 1), Neuro-Endocrine Tumor (NET; *n* = 1), and Thyroid Carcinoma (Thyr; *n* = 1). Most patients (71%) had not received systemic treatment before surgery (see patients characteristics in Supplementary Data 3). The percentage of immune cells expressing CD45 ranged from 0.7% to 95.7% among live cells (median 28.1%), with no significant difference observed across histological types (Fig. 1B). CD3 + T-cells varied across patients independently of tumor types, ranging from 10.8% to 88.1% (median 53.2%) among live CD45 + cells (Fig. 1C). The levels of CD8 + , CD4 + FoxP3-, and CD4 + FoxP3 + T-cell subsets varied between patients and among cancer histological subtypes (Fig. 1D-F). Overall, CD8 + , CD4 + FoxP3-, and CD4 + FoxP3 + T-cell levels were similar across cancer histological types, except for RCC, which showed significantly fewer CD4 + FoxP3 + T-cells than HNSCC (Fig. 1F). CD8 + T-cells (median 36.5%, range [1.9–87.3]) and CD4 + FoxP3- T-cells (38.6% [6.6–74.2]) were more abundant in the tumor microenvironment than CD4 + FoxP3 + T-cells (9.5% [0.1–40.6]), except for MM where no significant differences were observed between CD8 + and CD4 + FoxP3 + T-cells (Fig. 2A).

### Immune checkpoint protein expression is predominant on FoxP3^+^ CD4^+^ T-cells

Our primary analysis centred on the surface expression of 10 immune checkpoints (CD25, CD28, CD39, 4-1BB, CTLA-4, ICOS, OX40, PD-1, PD-L1, and TIGIT) on CD8 + , CD4 + FoxP3-, and CD4 + FoxP3 + T-cell subsets in 35 tumor specimens. Inter-individual variability was found for immune checkpoint proteins (ICPs), independently of cancer histological type. However, the ICP expression profile was homogeneous within T-cell subsets. CD4 + FoxP3 + T-cells were significantly more positive for ICPs than both CD4 + FoxP3- and CD8 + T-cells, with the exception of PD-1, which was more prevalent on CD4 + FoxP3 + than CD4 + FoxP3- T-cells, but not more than CD8 + cells (Fig. 2B). The ICPs CD25, CD28, CTLA-4, ICOS and OX40 were more frequently found on CD4 + FoxP3- than on CD8 + T-cells, although to a lesser extent than CD4 + FoxP3 + T-cells (Fig. 2B). Similar results were obtained by analyzing the level of ICP expression using mean fluorescence intensities (MFI), which correlates with protein abundance on cell membranes (Fig. 2C and Supplementary Data 4). CD39 and CD25 were the most differentially expressed ICPs between CD4 + FoxP3 + and CD4 + FoxP3- T-cell subsets (Fig. 2D). LAG3 was not part of our initial flow cytometry panel, but meanwhile, an anti-LAG3 checkpoint antibody (relatlimab) has been FDA/EMA approved. Therefore we decided to assess LAG3 expression in 3 additional tumor specimens (one HNSCC, one Hodgkin and one NSCLC). Overall we found a poor expression (< 4%) of LAG3 on T-cells membranes with a trend toward more expression on Tregs (median percentage expression: 0.87% of LAG3 on CD8 + , 0.49% on CD4 + Foxp3- and 3.8% on CD4 + Foxp3 +) (Supplementary Data 5).

Although the pattern of ICP expression was not associated with a specific tumor histology, we observed some trends. For example, in HNSCC, more CD8 + and CD4 + FoxP3- T-cells were CD25-positive than in RCC, and in NSCLC, more CD4 + FoxP3 + and CD4 + FoxP3- T-cells were CTLA-4-positive than in RCC (Supplementary Data 6).

### Immune checkpoint co-expression is predominant on FoxP3^+^ CD4^+^ T-cells

**Fig. 3:**
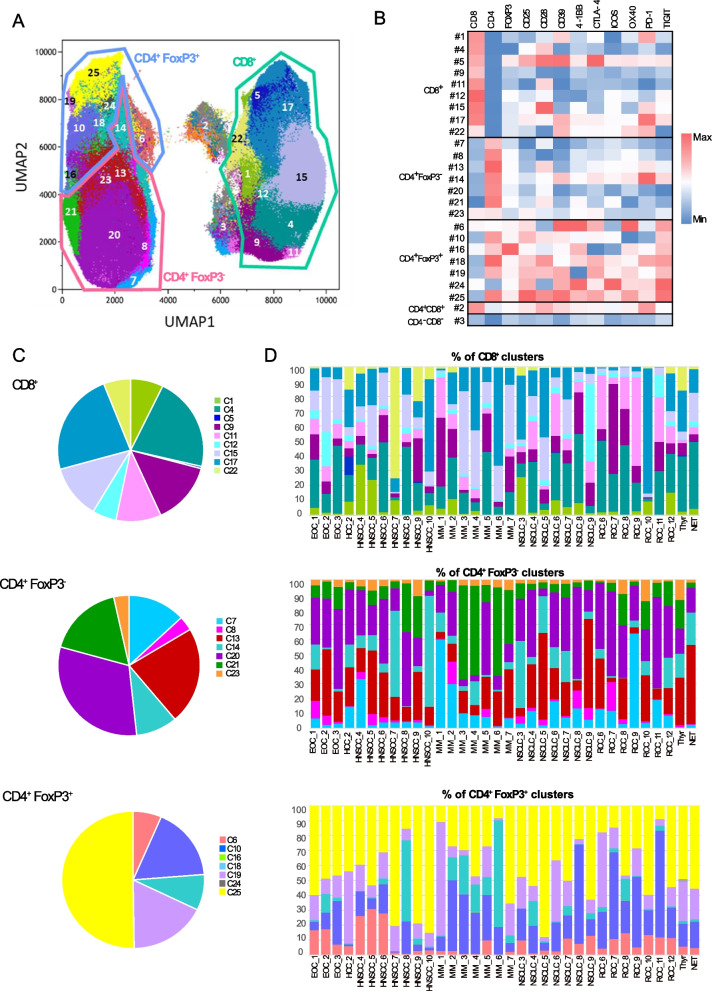
Unsupervised clustering of T-cell subsets according to the level of membrane protein expression. Unsupervised clustering analysis of flow cytometric dataset using PhenoGraph algorithm (*n* = 34). **A** UMAP displaying the 25 clusters defined based on the fluorescence intensity of each marker tested, including ICPs. **B** Heatmap showing the protein expression patterns in each cluster. Fluorescence intensity of each marker has been normalized independently. **C** Pie charts representing the relative abundance (mean) of each cluster in the whole cohort, in CD8^+^, CD4^+^FoxP3^−^ and CD4^+^FoxP3.^+^ T cells (left panels); stacked bar chart displaying the relative abundance of each cluster in each tumor specimen in the 3 T-cell subsets (right panels). ICPs: immune checkpoints: (CD25, CD28, CD39, 4-1BB, CTLA-4, ICOS, OX40, PD-1, PD-L1, and TIGIT)

We next analyzed our ICP phenotyping using the PhenoGraph algorithm [20] to characterize tumor-infiltrating T-cell subpopulations according to ICP co-expression. Nine clusters of CD8 + T-cells, seven clusters for each CD4 + FoxP3- and CD4 + FoxP3 + T-cells, one cluster of CD4 + CD8 + and one cluster of CD4-CD8- T-cells were identified (Fig. 3A). CD4 + FoxP3 + T-cell clusters exhibited patterns of multiple ICP co-expressions compared to CD8 + and CD4 + FoxP3- T-cell clusters (Fig. 3B). The distribution of clusters within the T-cell subsets were heterogeneous (Fig. 3C). In CD8 + T-cells, the most abundant cluster (#4) represented an average of 24.4% [1.9–48.1] and expressed none of the tested ICPs except for CD28, while the cluster expressing the most ICPs (#17) represented 19.9% [0.2–84.4] of CD8 + T-cells. In CD4 + FoxP3- T-cells, the most abundant cluster (#20) representing 29.5% [3.5–61.8], expressed none of the tested ICPs, while the most activated cluster (#14) accounted for only 12.9% [0.2–75.0] of CD4 + FoxP3- T-cells. In CD4 + FoxP3 + T-cells, the most abundant cluster (#25, 43.9% [8.2–88.0]) co-expressed all tested ICPs. We found a large interpatient variability, with none of the clusters showing a particular association with any histopathological tumor type (Fig. 3D). ICPs were more highly expressed in CD4 + FoxP3 + clusters, with cluster #25 (co-expressing all 9 ICPs), being the predominant CD4 + FoxP3 + cluster in 58.8% of tumors (Supplementary Data 7A and B, Fig. 3C and D). Interestingly, clusters #25 and #19 (CD4 + FoxP3 +) as well as clusters #5 and #22 (CD8 +) were significantly more abundant in HNSCC compared to RCC (Supplementary Data 8A). Comparison between tumors that were primary resected and those that were resected at relapse revealed a lower frequency of clusters co-expressing CD25, CD39, CTLA-4, OX40, and TIGIT (#6, #19) (Supplementary Data 8B). A classical supervised analysis, examining the proportion of cells double-positive for CD25 + and another checkpoint within CD4 + FoxP3 + cells (Supplementary Data 9A), or the proportion of FoxP3 + cells within CD4^+^ T-cells double-positive for CD25 + and another checkpoint (Supplementary Data 9B) confirmed the higher ICP co-expression in CD4 + FoxP3 + T-cells compared to CD4 + FoxP3- T-cells. Overall, this analysis showed that tumor infiltrating CD4 + FoxP3 + T-cells co-expressed most of our ICPs of interest.

We then attempted to determine whether the phenotypic profile of T-cells was associated with disease outcomes and prognostic factors such as lymph node metastasis, neutrophil-to-lymphocyte ratio (NLR), or derived neutrophil-to-lymphocyte ratio (dNLR), but no significant impact was found. However, a trend toward better overall survival was evident with a higher CD8 + /FoxP3 + ratio (Supplementary Data 10). It’s worth noting that this analysis was limited by the size of our cohort and the differences in prognosis across the different cancer histotypes.

### Intratumoral T-cell assessment using scRNA-Seq

**Fig. 4:**
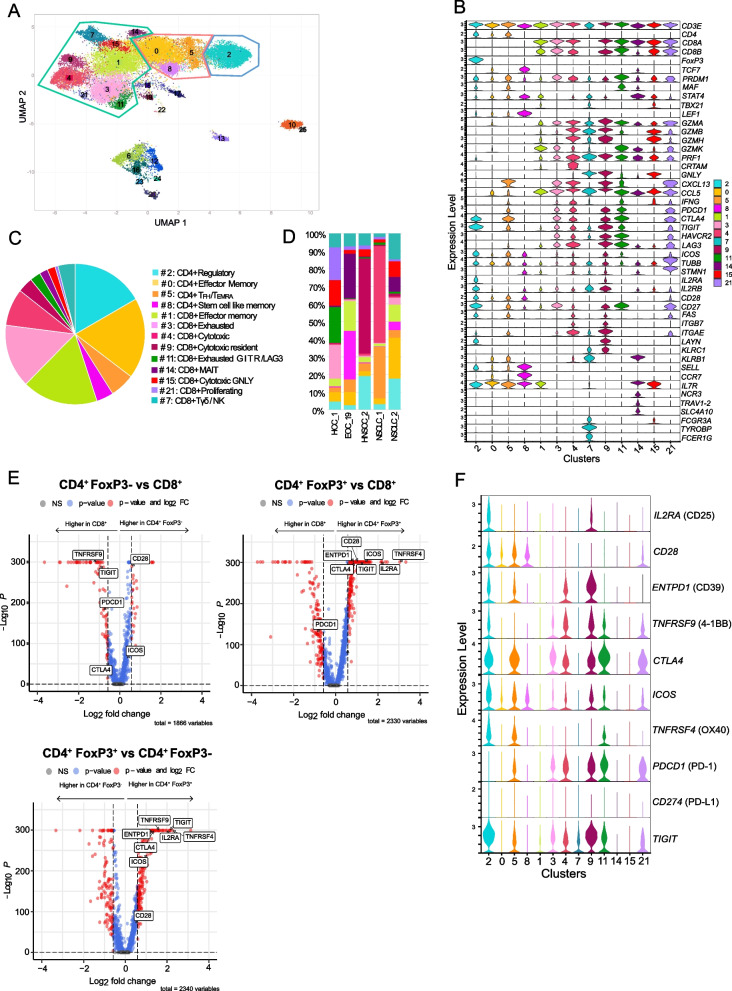
Unsupervised clustering of T-cell subsets according to the level of intracellular gene expression. Single-cell RNA sequencing of five fresh tumor specimens. **A** UMAP displaying clusters defined based on their gene expression profile. Created with Cerebro (R-studio^©^). The list of genes expressed by each cluster is provided in Supplementary Data 11. **B** Stacked violin plot displaying the expression distribution of selected cell markers in the T cell clusters. **C** Pie chart showing the relative abundance (mean) of each cluster in the whole cohort. **D** Stacked bar chart showing the relative abundance of each cluster in each tumor specimen. **E** Volcano plot displaying differential gene expression between *CD4*^+^*FOXP3*^−^ vs *CD8*^+^ T-cells (upper left panel); *CD4*^+^*FOXP3*^+^ vs *CD4*^+^*FOXP3*^−^T cells (bottom panel) and *CD4*^+^*FOXP3*^+^ vs *CD8*.^+^ T cells (upper right panel). Genes are plotted as log2 fold change versus the − log10 of the adjusted *p* value. Genes in red are significantly differentially expressed with a fold change > 1.5 compared to the reference population. **F**. Stacked violin plot displaying the expression distribution of ICPs in the T cell clusters. ICPs: immune checkpoints: (CD25, CD28, CD39, 4-1BB, CTLA-4, ICOS, OX40, PD-1, PD-L1, and TIGIT)

To understand the ICP expressions in T-cell subsets and their relationship with intratumoral clonality, we analyzed transcriptomes, including TCR sequences, in enriched CD45 + cells from five freshly resected tumors (2 NSCLCs, 1 HCC, 1 HNSCC, and 1 EOC) using droplet-based single-cell RNA sequencing (scRNA-Seq, 10X Genomics). We obtained the transcriptomic profile of 28,555 CD45 + cells, including 24,105 T-cells. After sequence alignment and quality control, we performed dimensional reduction analysis using UMAP (Fig. 4A) and assigned identified clusters to specific T-cell lineages according to the expression of canonical gene markers (Fig. 4B, Supplementary Data 11) [34–36]. We identified 9 clusters of CD8 + , 3 clusters of CD4 + FoxP3- and 1 cluster of CD4 + FoxP3 + T-cells (Fig. 4A and B).

The most abundant CD8 + clusters were effector memory CD8 + T-cells (#1: 17.5% [3.6–40.7]) as defined by the predominant co-expression of *IL7R, STAT4* and *GZMK*, and exhausted CD8 + T-cells (#3: 14.6% [0.7–40.9]) as defined by the predominant co-expression of *PD1, CTLA4*, *TIGIT, HAVCR2* (TIM3) and *LAG3* (see Supplementary Data 11). Other CD8 + clusters represented less than 10% each of the CD8 + cell lineage, such as cytotoxic CD8 + T-cells (#4: 8.5% [0.2–58.5]), CD8 + cytotoxic resident T-cells (#9: 3.5% [0.1–26.9]), exhausted *TNFRSF18* + *(GITR)*/*LAG3* + CD8 + T-cells (#11: 2.5% [0.1–7.4]), CD8 + MAIT (#14: 1.9% [0.0–6.7]), cytotoxic *GNLY* + CD8 + T-cells (#15: 1.9% [0.4–4.0]), and proliferating CD8 + T-cells (#21: 0.7% [0.1–1.9]). Cluster #7 consisted mainly of γδ T-cells or natural killer T-cells (NKT) cells (4.0% [0.9–5.3]) (Fig. 4C).

The most abundant CD4 + clusters were effector memory T-cells (#0: 18.6% [7.0–39.2]) as defined by the predominant expression of *IL7R* and regulatory T-cells (#2: 16.7% [6.0–45.9]) as defined by the predominant expression of *FOXP3*. The two other clusters of CD4 + cell lineage included follicular helper T-cells (T_FH_) and effector memory re-expressing CD45RA (T_EMRA_) (#5: 5.7% [2.2–20.8]) and stem cell like memory CD4 + T-cells (#8: 3.9% [0.1–14.9]) (Fig. 4C).

High heterogeneity was observed across tumors, as noted previously with flow cytometry staining (Fig. 4D). While some clusters were highly prevalent in all samples (> 5% in at least 4/5 samples), such as clusters #0, #1, #2, #5; #7; #15, others were over-represented in one tumor (Fig. 4D).

Next, we compared ICP gene expression between CD8 + , CD4 + FOXP3-, and CD4 + FOXP3 + clusters (Fig. 4E). Significantly differentially expressed ICPs were determined with an average Log2 fold-change (avg_log2FC) of > 0.58 or < -0.58 and a corresponding adjusted *p*-value < 0.05. *IL2RA* (CD25), *ENTPD1* (CD39), *CTLA4*, *ICOS*, *TNFRSF4* (OX40), and *TIGIT* expression was significantly higher in CD4 + FOXP3 + T-cells compared to both CD8 + and CD4 + FOXP3- T-cells. CD28 was more expressed by both subsets of CD4 + T-cells compared to CD8 + T-cells. *PDCD1* (PD-1) was less expressed by both subsets of CD4 + T-cells compared to CD8 + T-cells. *CD274* (PD-L1) mRNA was not detected in any T-cell subset. CD8 + T-cell subsets over-represented in one tumor expressed higher levels of ICPs (#3, #4, #9, #11, #21) (Fig. 4F). CD4 + cluster #5 had similar levels of *CD28*, *ENTPD1* (CD39), *CTLA4*, *ICOS*, and *CD274* (PD-L1) expression as CD4 + FOXP3 + cluster #2. Notably, cluster #2, the only cluster co-expressing CD4 and FOXP3 (Fig. 4B), co-expressed all ICP genes except PD-1 and PD-L1 (Fig. 4F).

### High expression of ICPs in clonally expanded *CD4*^+^*FOXP3*^+^ T-cells

**Fig. 5:**
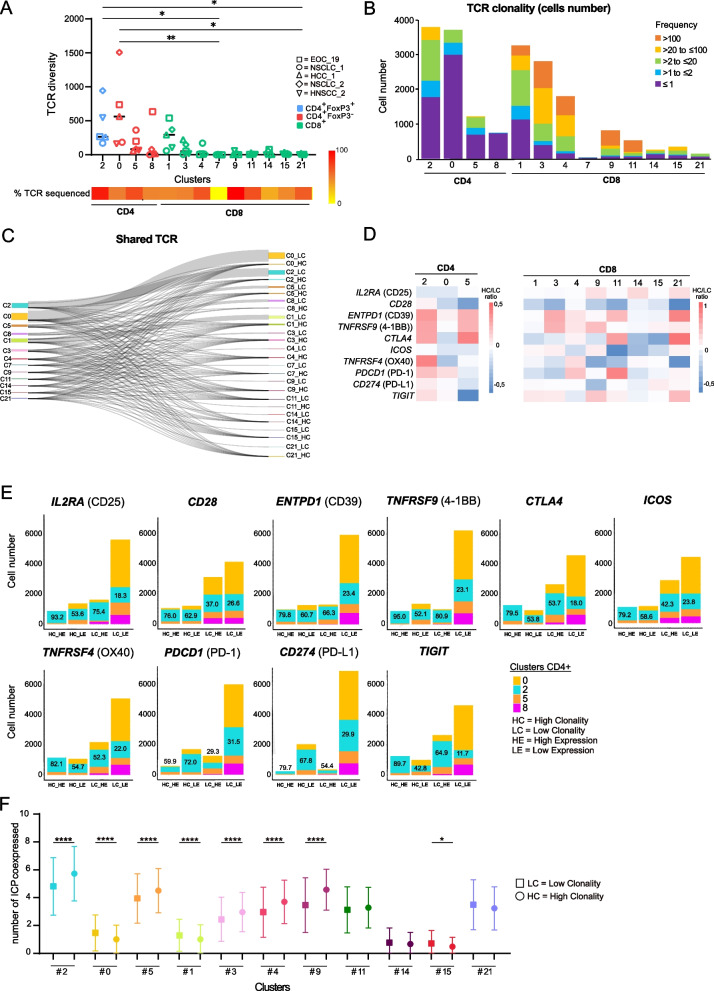
Immune checkpoints are more expressed by expanded clonotypes of intratumoral CD4^+^Foxp3^+^ T-cells. TCR repertoire analysis from the single-cell RNA sequencing dataset of five fresh tumor specimen. **A** TCR diversity showing the number of clonotypes per patient in each T cell cluster. Dunn’s multiple comparison test, *p value ≤ 0.05; **p value ≤ 0.01. **B** Stacked bar chart displaying the distribution of clonotype frequency in each cluster. **C** Sankey diagram showing clonotype sharing between clusters and according to clonality (LC ≤ 2 cells; HC > 2 cells). **D** Heatmap displaying differential ICP expression (median Log2 fold-change) between LC and HC T cells in each *CD4*^+^ (left panel) and *CD8*^+^ (right panel) clusters. **E** Stacked bar chart showing the distribution of T-cells from CD4^+^ clusters according to the level of ICP expression (above the median expression level (HE) or below the median expression level (LE)) and the expansion status (LC or HC). Median expression level was calculated independently for each sample and for each ICP. **F** Graph displaying the average number with standard deviation of ICP expressed per cells in LC and HC T cells for each cluster; Mann–Whitney test, *p value ≤ 0.05; **p value ≤ 0.01; ***p value ≤ 0.001; ****p value ≤ 0.0001.TCR: T cell receptor; LC: low clonality; HC: high clonality. ICPs: immune checkpoints: (CD25, CD28, CD39, 4-1BB, CTLA-4, ICOS, OX40, PD-1, PD-L1, and TIGIT)

We investigated the TCRαβ repertoire in five tumor specimens using scRNA-Seq. Excluding cluster #7 composed mainly of NKT-cells and γδ T-cells, 84.0% of cells assigned as T-cells had productive TCR sequences. The percentage ranged from 55.3% in cluster #14 to 97.3% in cluster #9 (Fig. 5A). We assessed TCR diversity, i.e., the number of unique TCRs, detecting 9309 different clonotypes in T-cell clusters. CD4 + T-cell clusters showed higher TCR diversity with 6993 clonotypes distributed among 10,833 CD4 + cells, compared to CD8 + clusters with 2524 clonotypes among 13,272 CD8 + cells (Fig. 5A). The highest diversity was found in cluster #0 (Effector Memory CD4 + T-cells), #1 (Effector Memory CD8 + T-cells), and #2 (Regulatory CD4 + T-cells).

TCR clonality analysis, which measures the number of T-cells expressing the same TCR or “clones”, revealed higher representation of expanded T-cell clones (≥ 3 cells expressing the same TCR subsequently to T-cell proliferation) in CD8 + clusters, with 72.2% of T-cells showing expanded clones, compared to 23.7% in CD4 + clusters (Fig. 5B). The most expanded clones, consisting of more than 100 T-cells expressing the same TCR, were found in CD8 + clusters (#1, #3, #4, #9, #11), while the highest rate of expanded clones among CD4 + clusters were observed in the *CD4* + *FOXP3* + cluster (#2). Although no TCRs were shared between patients, most likely due to MHC restrictions, 5.4% of detected clonotypes were distributed among clusters (Fig. 5C).

We then examined the relationship between ICP expression and TCR clonality. ICP gene expression was analyzed for each cluster, comparing high clonality (HC) to low clonality (LC) clonotypes. CD8 + clusters showed a wide range of ICP expression levels relative to clonality. All our checkpoints of interest, except *ICOS*, were expressed at higher levels in clonally expanding TCRs of *CD4* + *FOXP3* + (cluster #2). *ENTPD1* (CD39), *TNFRSF9* (4-1BB), and *CTLA4* were also expressed at higher levels in expanded clonotypes from the CD4 + T_FH_/T_EMRA_ (cluster #5), but not in the CD4 + Effector Memory (cluster #0). *TNFRSF4* (OX40) was upregulated in clonally expanding *CD4* + *FOXP3* + T-cells (cluster #2), but not in other clonally expanding CD4 + T-cells (cluster #0 & #5).

We classified T-cells into four compartments based on clonality and ICP expression levels: high clonality/high ICP expression (HC_HE), high clonality/low ICP expression (HC_LE), low clonality/high ICP expression (LC_HE), and low clonality/low ICP expression (LC_LE). Once again, high clonality was defined as having 3 or more T-cells with the same TCR, and high ICP expression was determined when exceeding the median ICP expression for a given patient. The HC_LE compartment contained the largest number of cells from CD8 + clusters, while the LC_LE compartment contained the largest number from CD4 + clusters (Fig. 5E and Supplementary Data 12). In the HC_HE compartment, an average of 88.5% of cells in CD8 + clusters belonged to overrepresented clusters within a given tumor (#3, #4, #9, #11, #21 (Supplementary Data 12)), while an average of 81.4% of CD4 + T-cells belonged to *CD4* + *FOXP3* + (cluster #2) (Fig. 5E).

We also investigated whether high clonality was associated with ICP co-expression and compared the expanded clones to the non-expanded ones. The highest average levels of ICP co-expression were observed in cluster #2 (Fig. 5F). Clusters predominantly found in one tumor (i.e. #3, #4, #9, #11, #21) tended to display higher levels of ICP co-expression compared to clusters found in all tumors except cluster #5 (i.e. #0, #1, #15). Significantly higher ICP co-expression was evident in expanded clonotypes from clusters #2 (mean 5.7 vs. 4.8), #5 (4.5 vs. 3.9), #3 (3.0 vs. 2.4), #4 (3.7 vs. 3.0), #9 (4.6 vs. 3.5) compared to non-expanded clonotypes. Conversely, expanded clonotypes from clusters #0 (1.0 vs. 1.5) and #1 (1.0 vs. 1.3) displayed lower levels of ICP co-expression.

## Discussion

Since the success of anti-cancer immunotherapies like anti-CTLA-4 and anti-PD-(L)1, many other ICP-targeted monoclonal antibodies have been developed with the goal of restoring anti-cancer T-cell responses. These mAbs were either agonistic or antagonistic for T-cell receptor checkpoints. However, except for anti-LAG3, none of them reached FDA or EMA approvals, primarily due to a lack of clinical efficacy. One reason for this setback could be a poor understanding of the actual expression levels of these therapeutic targets among T-cell subsets within the tumor microenvironment. In these clinical trials, the intratumoral assessment for target expression, target saturation, and target engagement upon treatment only relied on IHC staining and bulk transcriptomics. However, these methods couldn’t accurately estimate membrane protein expressions on the different subsets of tumor-infiltrating T-cells.

In this study, we assessed the expression profiles of 10 ICPs on intratumoral T-cells in freshly resected human primary tumors at the single cell level, exploring both the protein and transcriptomic levels of ICP expression. We observed that despite high inter-patient variability that was not histology-driven, ICP single- and co-expressions were significantly higher in CD4 + FoxP3 + T-cells compared to both CD8 + and CD4 + FoxP3- T-cells. On scRNAseq analysis, we found that ICPs were preferentially co-expressed in expanded *CD4* + *FOXP3* + T-cell clones.

In the sphere of immune checkpoint therapies, intratumoral PD-L1 expression levels measured by IHC are associated with treatment response and have been approved as companion diagnostic assays for anti-PD(L)1 therapy in several tumor indications [37]. However, it remains unclear if the predictive value of PD-L1 expression in the tumor microenvironment relies on its expression on immune, cancer cells, or both, and at the membrane or intracytoplasmic level [38, 39]. Hyperprogression, a paradoxical acceleration of cancer progression under immunotherapy, could be caused by PD-1 blockade of PD-1 + regulatory T-cells [40, 41]. The balance of PD-1 expression between effector T-cells and regulatory T-cells could predict the clinical efficacy of PD-1 blockade therapies, illustrating how ICP expression on specific subsets of intratumoral T-cells can impact their clinical activity [42].

ICP-targeted mAbs aim to stimulate CD8 + and CD4 + FoxP3- anti-tumor T-cells while inhibiting immunosuppressive CD4 + FoxP3 + regulatory T-cells. These mAbs target co-inhibitory ICPs like TIGIT and CD39 or co-stimulatory ICPs like OX40, 4-1BB, and ICOS, all thought to be mainly expressed by anti-tumor effector T-cells [2]. Here, we found unexpectedly that such ICP molecules were highly and predominantly expressed by CD4 + FoxP3 + T-cells in primary solid tumors. Recently, Szeponik et al. showed that, in human colorectal tumors, CD39 is mostly expressed by intratumoral CD4 + Foxp3 + cells [43]. Here we show that this observation is not limited to CD39 and is not tumor histology dependent.

CD39 is also a marker of tumor-specific/reactive CD4 + and CD8 + effector T-cells in the tumor microenvironment [44–47]. In our study, the presence of CD39 + T-cells was variable, revealing that in early-stage lesions (either primary tumors or local relapses eligible for surgical intervention), tumor-reactive T-cell responses are heterogeneous. In *CD8*^+^ clusters, *ENTPD1* (CD39) was more expressed in T-cells with high clonality (HC) compared to T-cells with low clonality (LC), but only in certain clusters over-represented in a given tumor i.e. clusters #3, #4, #11, #21 (Fig. 5D). Also, we observed that CD39 was more expressed in HC than in LC in the shared cluster of *CD4*^+^*FOXP3*^+^ T-cells (cluster #2) suggesting that CD39 could also be a marker of tumor-specific regulatory T-cells. At the protein level, CD39 was highly expressed by CD4 + FoxP3 + T-cells (82.2% [10.6–99.3]), as previously observed [45, 48, 49]. Moreover, CD25 was only expressed by the *FOXP3* + transcriptomic cluster within CD4 + cells. In the context of murine models with OVA expressing tumors, Marabelle et al. showed that many intratumoral FoxP3^+^ T-cells are bystanders and that only the ones with tumor cell antigen reactivity upregulate ICPs such as OX40 and CTLA-4 [50]. Of note, the membrane co-expression of CD25 and CD39 in CD4^+^ T-cells allowed us to detect a median 66.8% [2.5–95.9] of FoxP3^+^ cells, suggesting that their co-expression could offer an option to identify Tregs when FoxP3 intracellular staining is not feasible (e.g. flow cytometry applied to fresh tumor biopsies in the context of clinical trials).

The comparison of methods to assess gene and protein expression levels revealed that ICP RNA expression cannot always be extrapolated to the protein level. The assessment of ICP expression using 10X genomics scRNA-Seq could sometimes be misleading, as observed with *CTLA-4*, *ICOS* and *4-1BB* genes, which though highly expressed at the gene level were minimally expressed at the protein level.

In this study, we correlated immunological data with clinical data collected from patient medical records. However, we found no significant correlation between the analyzed parameters and disease outcome. This is possibly due to the small size of our cohort and the heterogeneity of the cancers enrolled. Nevertheless, we observed that certain subsets of intratumoral CD4 + FoxP3 + T-cells may be more abundant in primary tumors than in relapsing tumors. Furthermore, we noted that the proportions of tumor-reactive CD8 + and CD4 + FoxP3- T-cells were similar, except for MM, which had significantly fewer tumor-reactive CD4 + FoxP3- T-cells than in NSCLC. Notably, all MM samples came from loco-regional lymph node metastatic relapses, while our NSCLC samples came from primary lung tumors, suggesting that tumor-reactive T-cell responses may be more prevalent in primary tumors than in relapsing/metastatic lesions. This observation aligns with previous studies that showed metastatic lesions less infiltrated by T-cells than in their paired primary tumors [51, 52].

Our results should be analyzed in their technical and clinical contexts. First, as opposed to IHC stainings, flow cytometry can clearly distinguish between membrane and intracellular protein expression and specify by which subset of cells these proteins are expressed. Single-cell transcriptomics captures the predominant genes expressed by single cells, but the RNA expression does not necessarily correlate with protein expression. However, flow cytometry and single cell transcriptomics cannot describe the geographical distribution of those immune cells within tumors, nor quantify the level of interactions between cells in the TME. Second, although we appreciate the logistical complexity of prospectively processing fresh tumor samples, our results would benefit from confirmation within larger cohorts to account for the important variability observed between patients and the potential impact of tumor histology on the proportions and/or phenotype of immune cells. Finally, our cohort was mostly made of primary resected tumors and we do not know whether our results would still hold for local or distant metastases.

Anti-immune checkpoint drugs are still under active clinical investigation in metastatic cancer patients and are now also being developed in the neo-adjuvant setting for patients with localized tumors [53–56]. The high inter-individual variability that we have found in terms of proportions of immune cells and membrane immune checkpoint protein levels by flow cytometry should affect the efficacy of such immune checkpoint targeted therapies. Therefore, such flow cytometry methods on fresh tumors should be incorporated during screening periods of clinical trials to rapidly assess intra-tumor target expressions, facilitating patient stratification in enrollment, ultimately reducing the high failure rates within drug development in oncology. Also, we found that the ICPs tested in this study, i.e., CD25, CD28, CD39, 4-1BB, CTLA-4, ICOS, OX40, PD-1, PD-L1 and TIGIT, were predominantly co-expressed by CD4 + FoxP3 + T-cells, which by single-cell TCR sequencing represented the majority of clonally expanding T-cells in the TME of primary human solid tumors. These results provide the rationale for developing drugs to either selectively deplete deleterious tumor-specific CD4 + FoxP3 + T-cells, or to reposition certain agonistic oncology drugs (e.g. anti-OX40) in the field of autoimmune diseases and transplantation, should they indeed boost CD4 + FoxP3 + Treg-cells. Overall, our data suggest that neo-adjuvant trials testing novel anti-checkpoint antibodies for localized primary tumors should first select eligible patients by checking the expression of the corresponding immune checkpoint using flow cytometry on baseline fresh tumor biopsies.

### Supplementary Information

**Additional file 1: Supplementary Data 1.** Immune checkpoint targeted monoclonal antibodies assessed in early phase oncology trials.

**Additional file 2: Supplementary Data 2.** Impact of the dissociation procedure on immune checkpoint expression assessed by flow cytometry. Tumor specimens were divided into 3 pieces, the first one was dissociated using our routine procedure (#1 dissociation of reference, 75 minutes, 37°C, with enzymes), the second one was dissociated in the conditions of reference (75 min, 37°C) but without enzymes, the last one was dissociated mechanically (15 min, room temperature, no enzyme). An additional condition consisted in analyzing independently the cells that were released spontaneously in the supernatant prior dissociation (Fig. 3). ICP expression was assessed using flow cytometry. (A) Percentage of immune checkpoint protein (ICP) positive cells in CD8^+^, CD4^+^FoxP3^-^ and CD4+FoxP3+ T cells from 5 tumor specimens. (B) Mean fluorescence intensity of ICPs in CD8^+^, CD4^+^FoxP3^-^ and CD4^+^FoxP3^+^ T cells from 5 tumor specimens.

**Additional file 3: Supplementary Data 3.** Patient characteristics.

**Additional file 4: Supplementary Data 4.** Fluorescence intensity detected for each ICP. Histograms displaying representative fluorescence intensity of each ICP tested for each T-cell subset.

**Additional file 5: Supplementary Data 5.** LAG3 expression in CD8^+^, CD4^+^FoxP3^-^ and CD4^+^FoxP3^+^ T-cells. LAG3 expression was assessed by flow cytometry using the clone C11C365 (Biolegend, 369308) in three tumor specimens.

**Additional file 6: Supplementary Data 6.** Immune checkpoint expression in the tumor microenvironment across histopathological types. Percentage of ICP positive cells in CD8^+^, CD4^+^FoxP3^-^ and CD4^+^FoxP3^+^ T cells from 35 tumor specimens in the different histologies. The red dotted line delineates the median of the whole cohort.

**Additional file 7: Supplementary Data 7.** Expression levels of ICPs and proportion of ICP-positive cells across clusters. (A) Mean Fluorescence intensity of ICPs in each tumor for each cluster. (B) Percentage of ICP-positive cells among CD3^+^ T-cells in each tumor for each cluster. The black line indicates the mean.

**Additional file 8: Supplementary Data 8.** Distribution of intratumoral T-cell clusters according to tumor types and relapsing status. (A) T-cell cluster frequency according to tumor types (*n*=31). Dunn’s multiple comparison test, *p value ≤ 0.05; **p value ≤ 0.01; ***p value ≤ 0.001; ****p value ≤ 0.0001. (B) T-cell cluster frequency in tumors analyzed at the time of primary or relapsing tumor resection (*n*=34). Mann-Whitney test, *p value ≤ 0.05; **p value ≤ 0.01; ***p value ≤ 0.001; ****p value ≤ 0.0001.

**Additional file 9: Supplementary Data 9.** CD25 and other ICP co-expression to detect intratumoral regulatory T-cells. (A) Gating strategy of flow cytometry analyses performed in 35 tumors to assess ICP co-expression and percentages of double-ICP positive CD4^+^ T cells within FoxP3^-^ and FoxP3^+^ subsets. (B) Gating strategy of flow cytometry analyses performed in 35 tumors to assess ICP co-expression and percentages of FoxP3^-^ and FoxP3^+^ T cells within double-ICP positive CD4^+^ T cells. Mann-Whitney test, *p value ≤ 0.05; **p value ≤ 0.01; ***p value ≤ 0.001; ****p value ≤ 0.0001.

**Additional file 10: Supplementary Data 10.** Impact of CD8^+^/FoxP3^+^ ratio, metastatic lymph node invasion, NLR and dNLR on overall survival. Kaplan-Meier curves displaying the overall survival starting at the date of the surgery according to (A) CD8^**+**^/FoxP3^**+**^ ratio, (B) metastatic lymph node invasion, (C) NLR and (D) dNLR. NLR: neutrophil-to-lymphocyte ratio; dNLR:derived neutrophil-to-lymphocyte ratio.

**Additional file 11: Supplementary Data 11.** Assignment of single-cell clusters to T-cell lineages according to the expression of canonical gene markers.

**Additional file 12: Supplementary Data 12.** Distribution of *CD8*^+^ T cells according to ICP expression level and clonality. Stacked bar chart showing the distribution of T cells from *CD8*^+^ clusters according to the level of ICP expression (above the median expression level (HM) or below the median expression level (LM)) and the expansion status (LC or HC). Median expression level was calculated independently for each sample and for each ICP.

## Data Availability

The datasets used and/or analyzed during the current study are available from the corresponding author on reasonable request.

## Abbreviations

CTLA-4: Cytotoxic T-Lymphocyte Associated Protein 4
EMA: European Medicines Agency
ENTPD1: Ectonucleoside Triphosphate Diphosphohydrolase 1
EOC: Epithelial Ovarian Cancer
FDA: Food and Drug Administration
FoxP3: Forkhead box P3
HC: High Clonality
HCC: HepatoCarcinoma
HE: High Expression
HNSCC: Head and Neck Squamous Cell Carcinoma
HVG: Highly Variable Genes
ICOS: Inducible T-cell COStimulator
ICP: Immune CheckPoint
IHC: ImmunoHisto Chemestry
LC: Low Clonality
LE: Low Expression
mAbs: Monoclonal Antibodies
MFI: Mean Fluorescence Intensity
MHC: Major Histocompatibility Complex
MM: Mestatatic Melanoma
NET: Neuro-Endocrine Tumor
NSCLC: Non-Small Cell Lung Cancer
PD-1/PDCD1: Programmed Cell Death 1
PD-L1: Programmed Cell Death Ligand 1
RCC: Renal Cell Carcinoma
scRNA-Seq: Single-cell RNA sequencing
TCR: T-cell Receptor
Thyr: Thyroid Carcinoma
TIGIT: T Cell Immunoreceptor With Ig And ITIM Domains
TNFRSF4: TNF Receptor Superfamily Member 4
TNFRSF9: TNF Receptor Superfamily Member 9
UC: Urothelial Carcinoma
