# Supplementary material for: Immune checkpoints are predominantly co-expressed by clonally expanded CD4+FoxP3+ intratumoral T-cells in primary human cancers
Source: J Exp Clin Cancer Res. 2023 Dec 6;42:333. doi: 10.1186/s13046-023-02897-6 (PMC10699039; doi:10.1186/s13046-023-02897-6)
Supplement: Supplementary file 1 — Additional file 1: Supplementary Data 1. Immune checkpoint targeted monoclonal antibodies assessed in early phase oncology trials. [file 13046_2023_2897_MOESM1_ESM.pdf]

Supplementary Data 1

TARGET	COMPOUND	Mab type	COMPANY	TRIAL	ORR	REF
OX40	INCAGN01949	agonistic IgG1K	Incyte	NCT02923349	1/87 (1,1%)	Davis EJ, et al. J Immunother Cancer. 2022;10:e004235.
	Ivuxolimab	agonistic IgG2	Pfizer	NCT02315066	3/52 (5,8%)	Diab A, et al. Clin Cancer Res. 2022;28:71–83.
	GSK3174998	agonistic IgG1	GSK	NCT02528357	1/45 (2%)	Postel-Vinay S, et al. Cancer Res. 2020;80:CT150–CT150.
	MOXR0916	agonistic IgG1	Genentech	NCT02219724	2/172 (0%)	Kim TW, et al. Clin Cancer Res. 2022;28:3452–63.
	MEDI6469	murine agonistic IgG1	Medimmune/AZ	NCT01644968	0/27 (0%)	Curti BD, et al. Cancer Res. 2013;73:7189–98.
	BMS-986178	agonistic IgG1	BMS	NCT02737475	0/20 (0%)	Gutierrez M, et al. Clin Cancer Res. 2021;27:460–72.
	MEDI0562	agonistic IgG1K	Medimmune/AZ	NCT02318394	2/50 (4%)	Glisson BS, et al. Clin Cancer Res. 2020;26:5358–67.
4-1BB	Utomilumab	agonistic IgG2	Pfizer	NCT01307267	2/53 (4%)	Segal NH, et al. Clin Cancer Res. 2018;24:1816–23.
	Urelumab	agonistic IgG4	BMS	NCT01775631	6/60 (10%)	Timmerman J, et al. Am J Hematol. 2020;95:510–20.
	AGEN2373	agonistic IgG1	Agenus	NCT04121676	0/19 (0%)	Tolcher AW, et al. J Clin Oncol. 2021;39:2634.
ICOS	feladilimab	agonistic IgG4	GSK	NCT02723955	2/13 (8%)	Balar AV, et al. J Clin Oncol. 2021;39:4519.
	vopratelimab	agonistic IgG1	Jounce Therapeutics	NCT02904226	1/70 (1,4%)	Yap TA,et al. Clin Cancer Res. 2022;28:3695–708.
TIGIT	tiragolumab	antagonistic IgG1K	Genentech	NCT02794571	0/73 (0%)	Bendell JC, et al. Cancer Res. 2020;80:CT302–CT302.
	vibostolimab	antagonistic IgG1K	MSD	NCT02964013	0/76 (0%)	Niu J, et al. Ann Oncol. 2022;33:169–80.
