# Supplementary figures and images for: Immune checkpoints are predominantly co-expressed by clonally expanded CD4+FoxP3+ intratumoral T-cells in primary human cancers
Source: J Exp Clin Cancer Res. 2023 Dec 6;42:333. doi: 10.1186/s13046-023-02897-6 (PMC10699039; doi:10.1186/s13046-023-02897-6)

# Supplementary Data 2

A

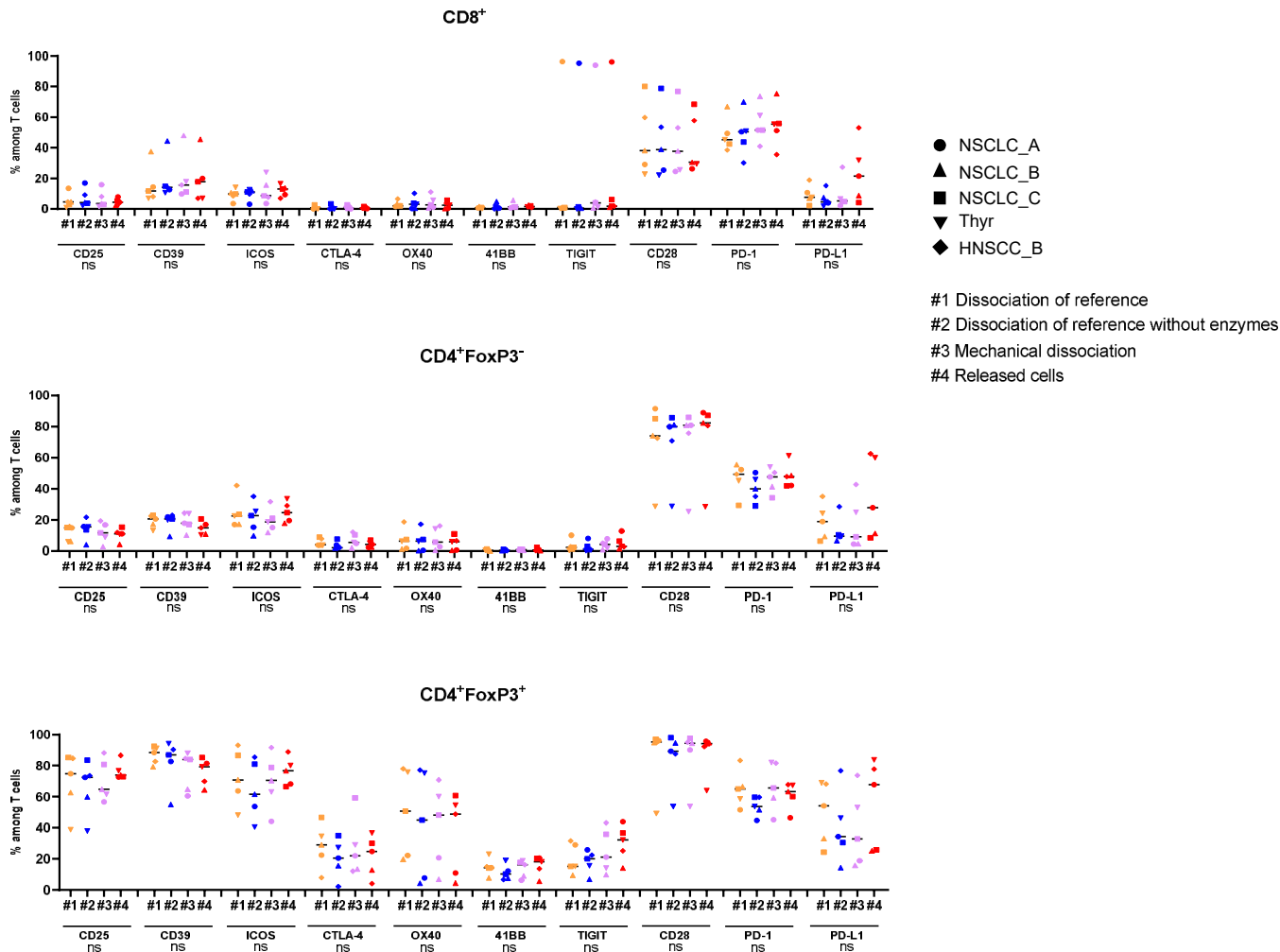

B

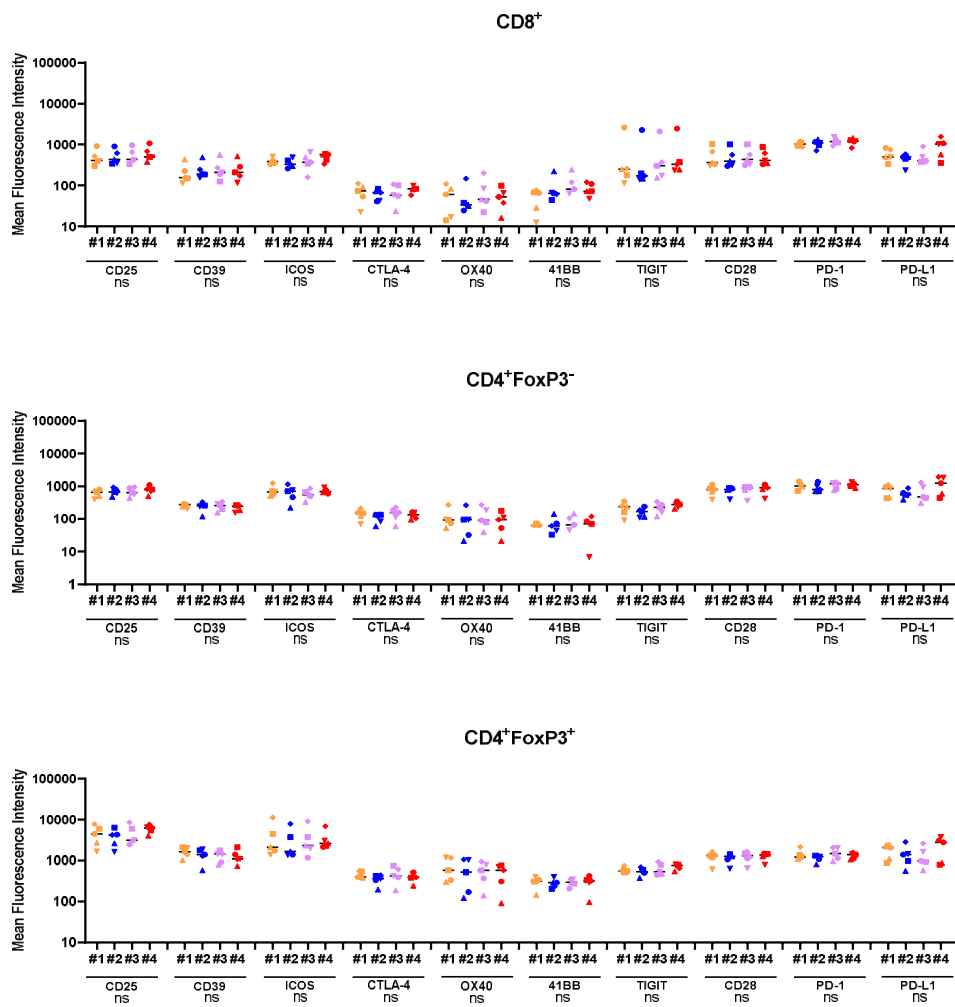

Supplement: Supplementary file 2 — Additional file 2: Supplementary Data 2. Impact of the dissociation procedure on immune checkpoint expression assessed by flow cytometry. Tumor specimens were divided into 3 pieces, the first one was dissociated using our routine procedure (#1 dissociation of reference, 75 minutes, 37°C, with enzymes), the second one was dissociated in the conditions of reference (75 min, 37°C) but without enzymes, the last one was dissociated mechanically (15 min, room temperature, no enzyme). An additional condition consisted in analyzing independently the cells that were released spontaneously in the supernatant prior dissociation (Fig. 3). ICP expression was assessed using flow cytometry. (A) Percentage of immune checkpoint protein (ICP) positive cells in CD8+, CD4+FoxP3- and CD4+FoxP3+ T cells from 5 tumor specimens. (B) Mean fluorescence intensity of ICPs in CD8+, CD4+FoxP3- and CD4+FoxP3+ T cells from 5 tumor specimens. [file 13046_2023_2897_MOESM2_ESM.pdf]

# Supplementary Data 4

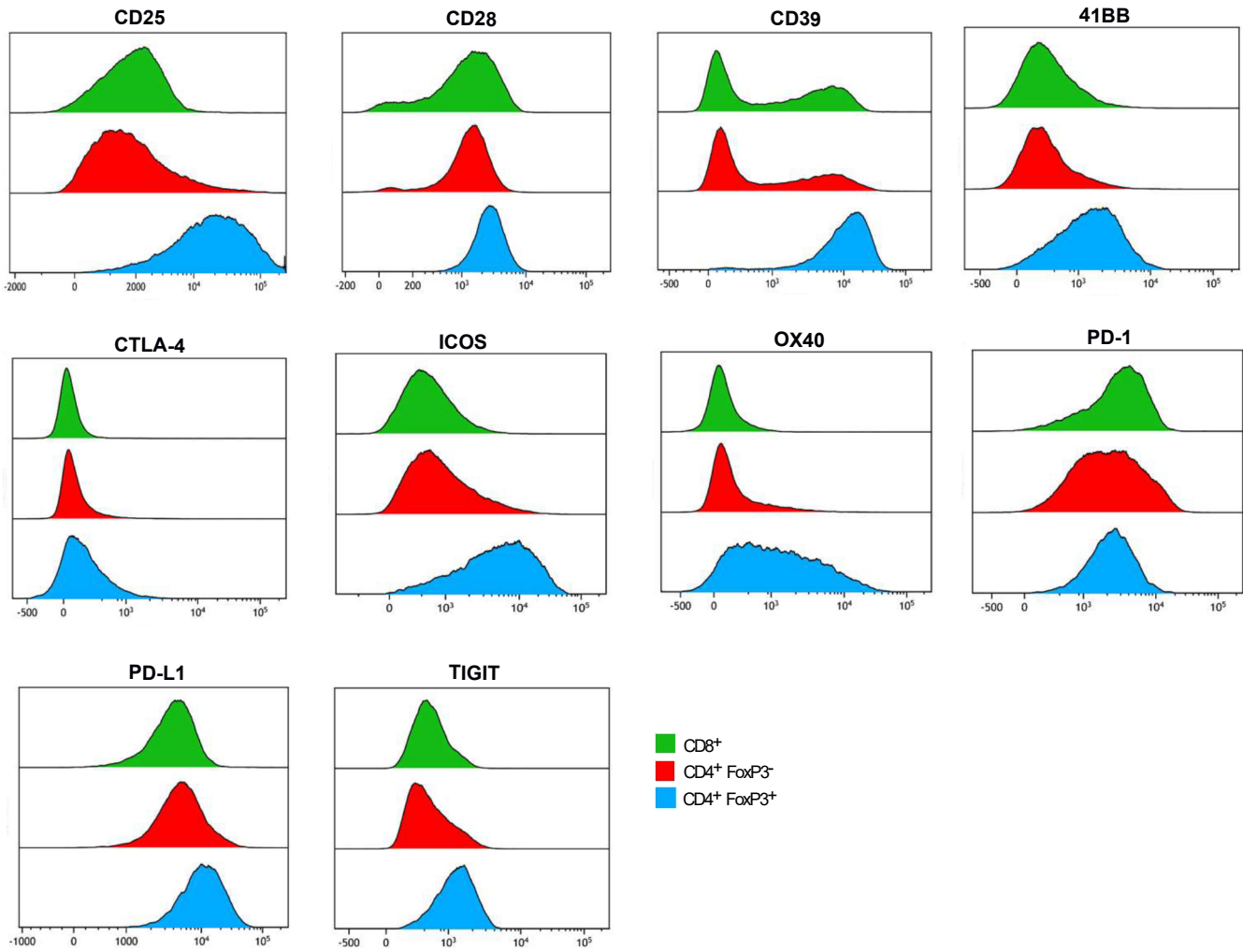

Supplement: Supplementary file 4 — Additional file 4: Supplementary Data 4. Fluorescence intensity detected for each ICP. Histograms displaying representative fluorescence intensity of each ICP tested for each T-cell subset. [file 13046_2023_2897_MOESM4_ESM.pdf]

# Supplementary Data 5

LAG3

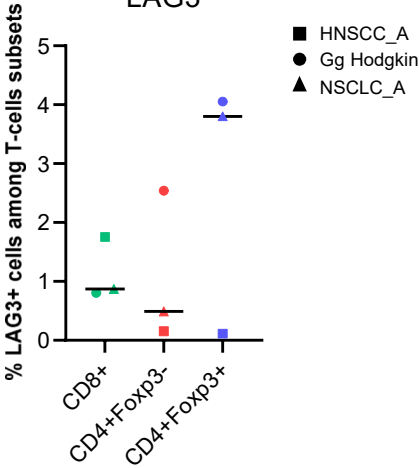

Supplement: Supplementary file 5 — Additional file 5: Supplementary Data 5. LAG3 expression in CD8+, CD4+FoxP3- and CD4+FoxP3+ T-cells. LAG3 expression was assessed by flow cytometry using the clone C11C365 (Biolegend, 369308) in three tumor specimens. [file 13046_2023_2897_MOESM5_ESM.pdf]

Supplementary Data 6

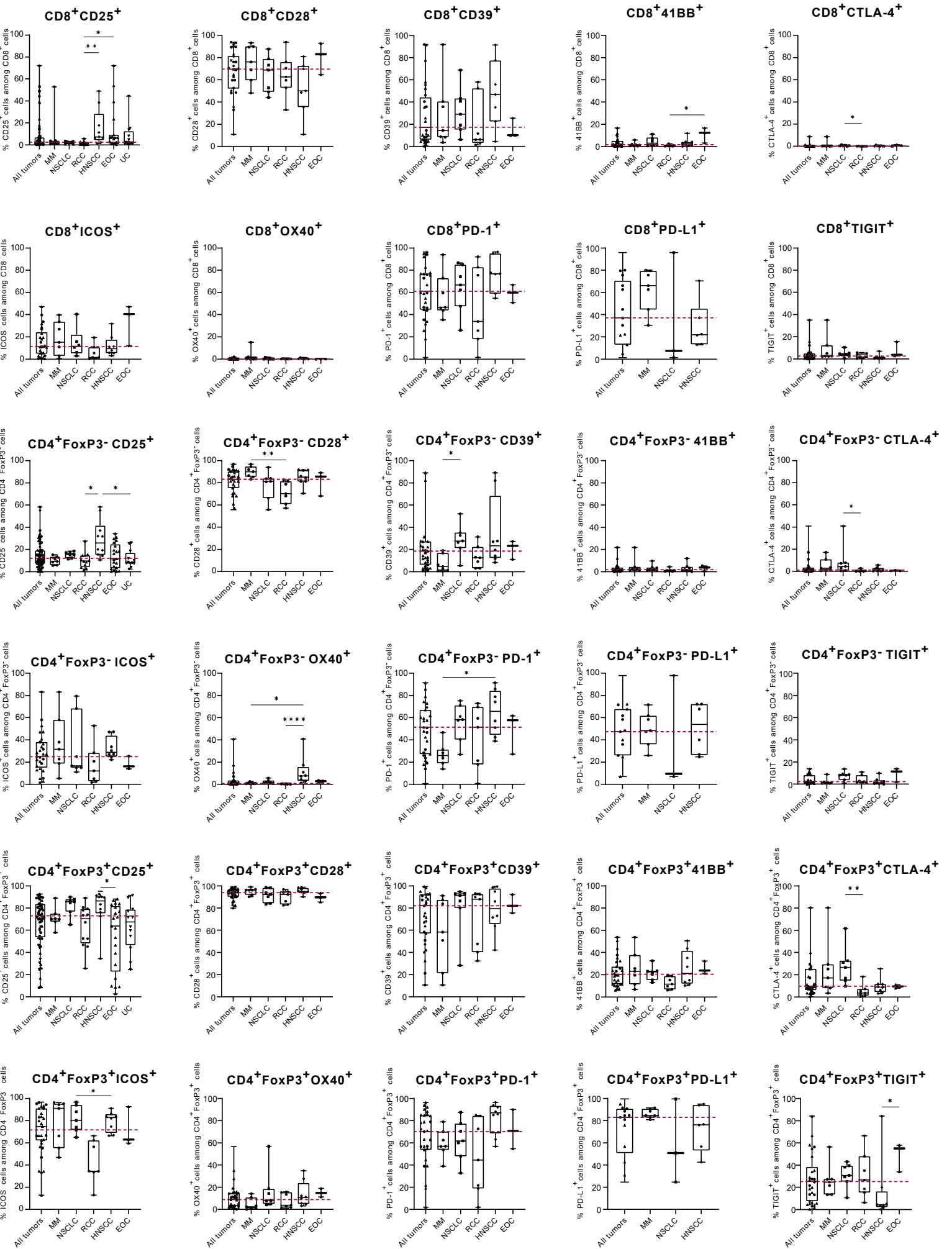

Supplement: Supplementary file 6 — Additional file 6: Supplementary Data 6. Immune checkpoint expression in the tumor microenvironment across histopathological types. Percentage of ICP positive cells in CD8+, CD4+FoxP3- and CD4+FoxP3+ T cells from 35 tumor specimens in the different histologies. The red dotted line delineates the median of the whole cohort. [file 13046_2023_2897_MOESM6_ESM.pdf]

# Supplementary Data 7

CD8<sup>+</sup>  
CD4<sup>+</sup> FoxP3<sup>-</sup>  
CD4<sup>+</sup> FoxP3<sup>+</sup>

A

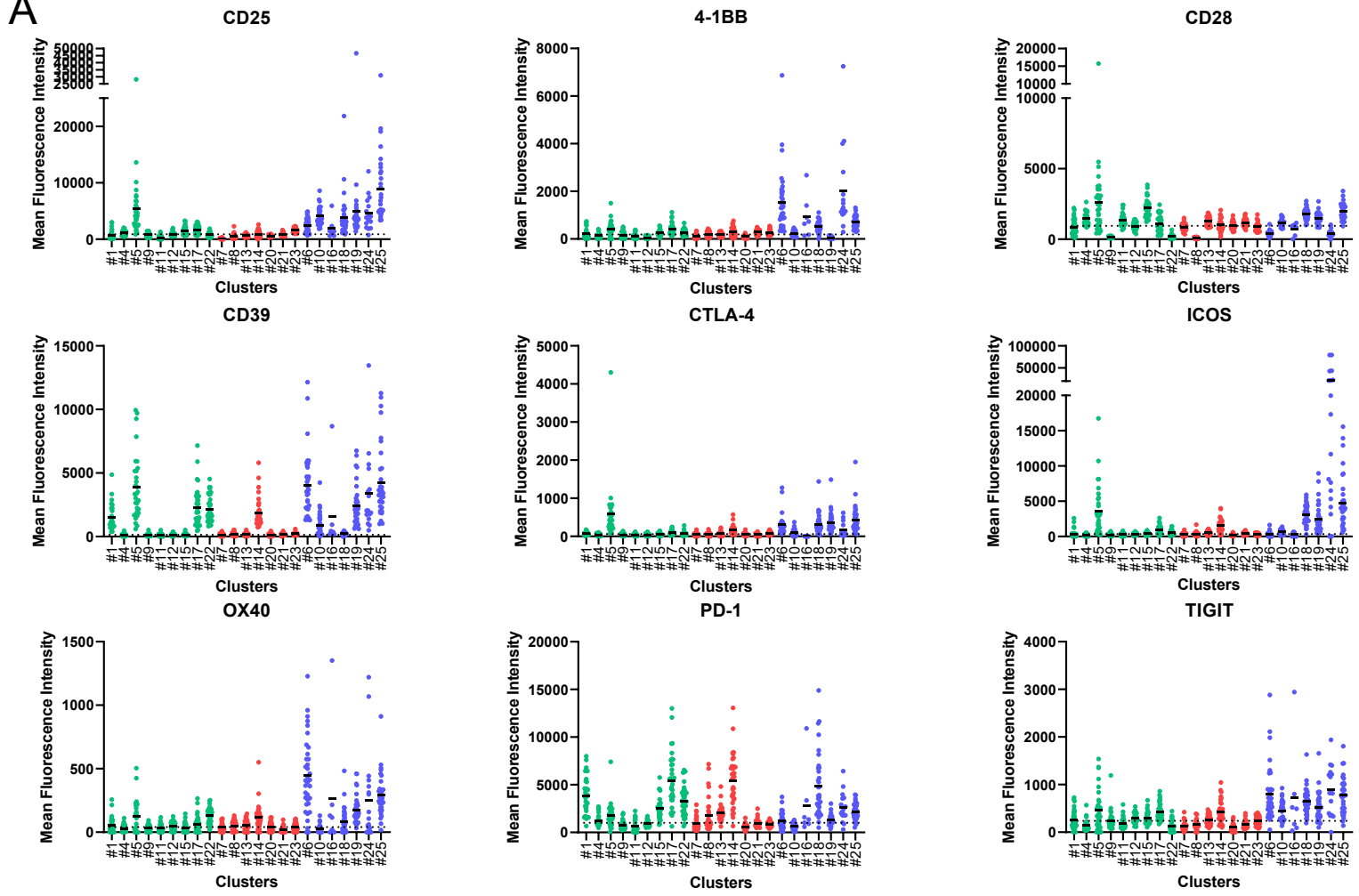

B

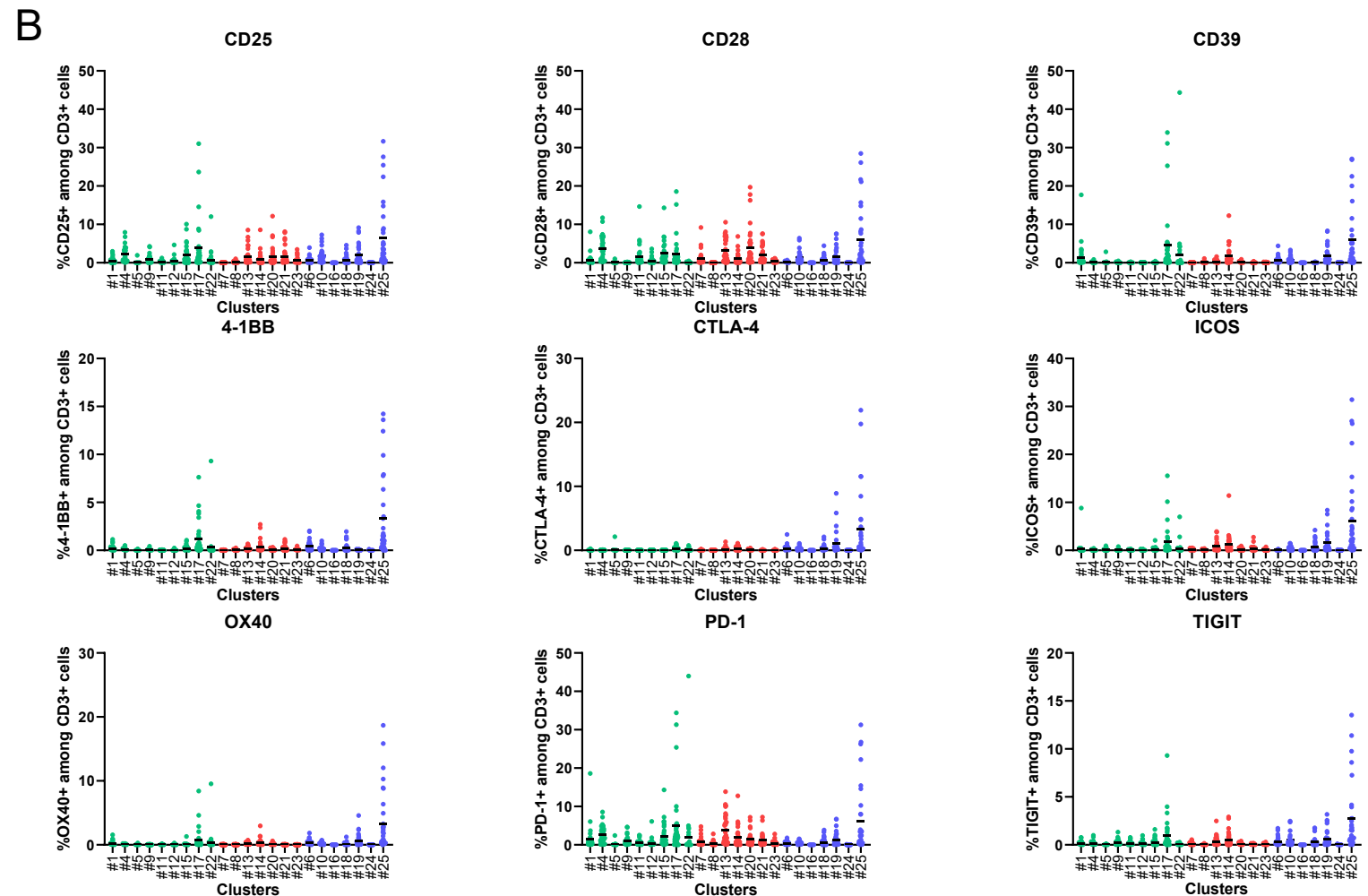

Supplement: Supplementary file 7 — Additional file 7: Supplementary Data 7. Expression levels of ICPs and proportion of ICP-positive cells across clusters. (A) Mean Fluorescence intensity of ICPs in each tumor for each cluster. (B) Percentage of ICP-positive cells among CD3+ T-cells in each tumor for each cluster. The black line indicates the mean. [file 13046_2023_2897_MOESM7_ESM.pdf]

# Supplementary Data 8

A

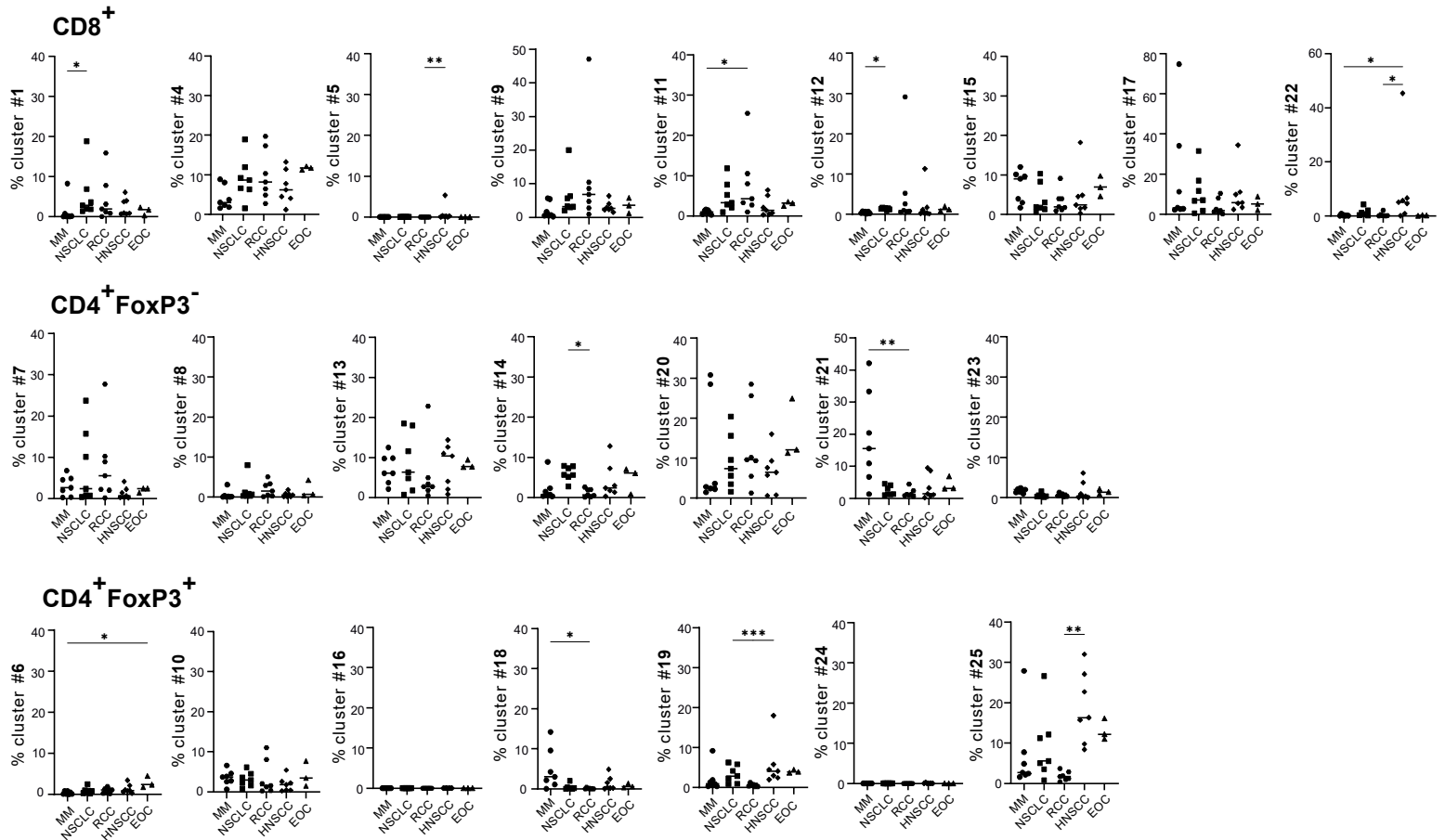

B

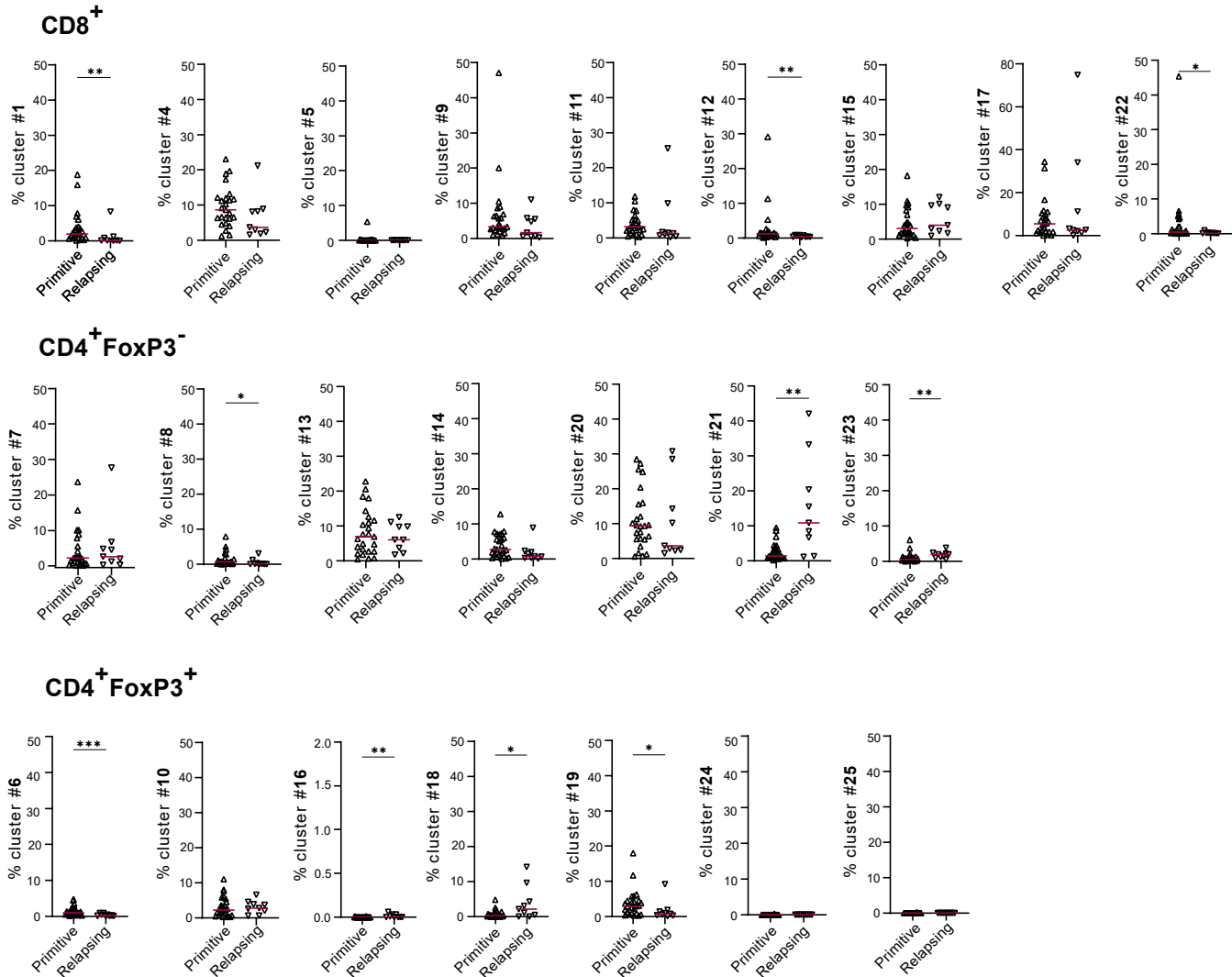

Supplement: Supplementary file 8 — Additional file 8: Supplementary Data 8. Distribution of intratumoral T-cell clusters according to tumor types and relapsing status. (A) T-cell cluster frequency according to tumor types (n=31). Dunn’s multiple comparison test, *p value ≤ 0.05; **p value ≤ 0.01; ***p value ≤ 0.001; ****p value ≤ 0.0001. (B) T-cell cluster frequency in tumors analyzed at the time of primary or relapsing tumor resection (n=34). Mann-Whitney test, *p value ≤ 0.05; **p value ≤ 0.01; ***p value ≤ 0.001; ****p value ≤ 0.0001. [file 13046_2023_2897_MOESM8_ESM.pdf]

Supplementary Data 9

A

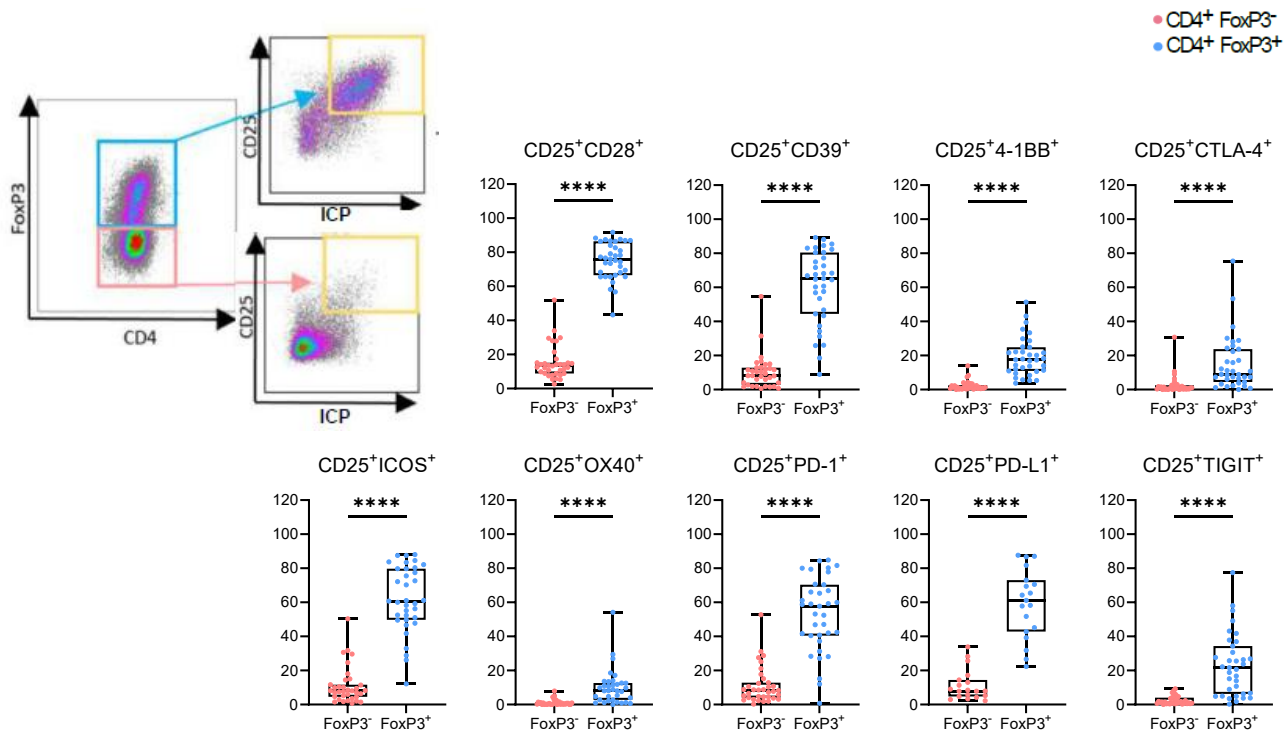

B

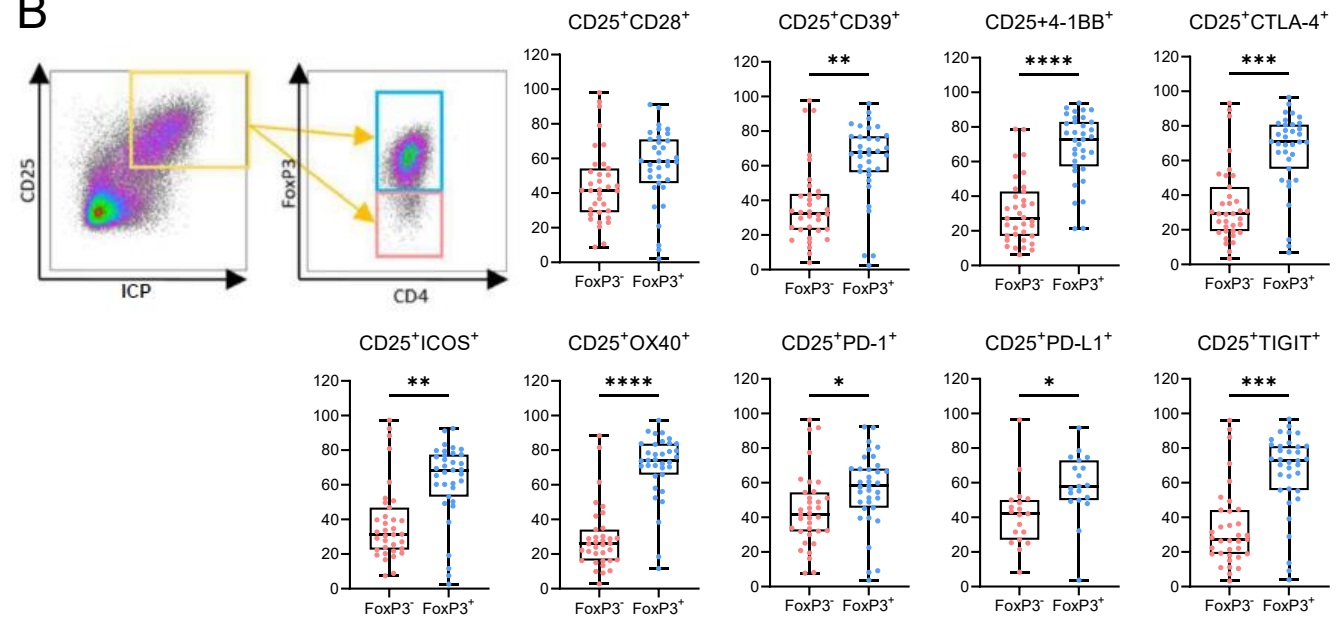

Supplement: Supplementary file 9 — Additional file 9: Supplementary Data 9. CD25 and other ICP co-expression to detect intratumoral regulatory T-cells. (A) Gating strategy of flow cytometry analyses performed in 35 tumors to assess ICP co-expression and percentages of double-ICP positive CD4+ T cells within FoxP3- and FoxP3+ subsets. (B) Gating strategy of flow cytometry analyses performed in 35 tumors to assess ICP co-expression and percentages of FoxP3- and FoxP3+ T cells within double-ICP positive CD4+ T cells. Mann-Whitney test, *p value ≤ 0.05; **p value ≤ 0.01; ***p value ≤ 0.001; ****p value ≤ 0.0001. [file 13046_2023_2897_MOESM9_ESM.pdf]

# Supplementary Data 10

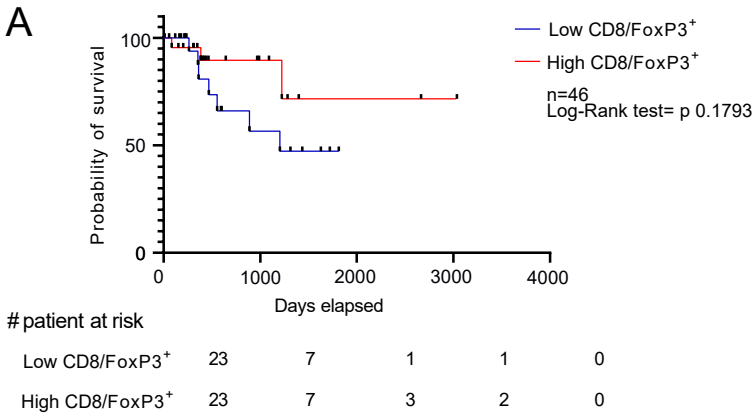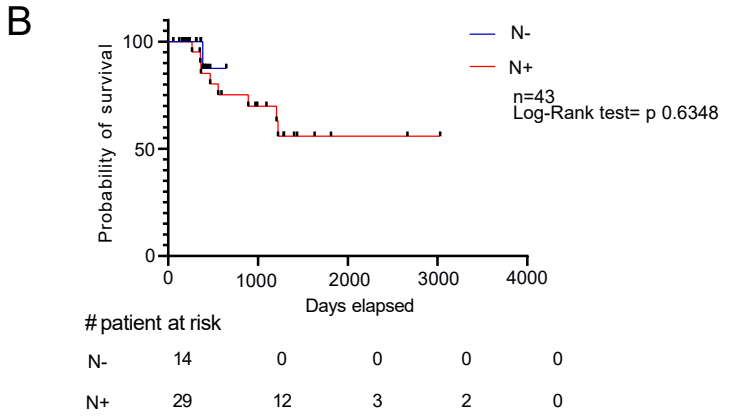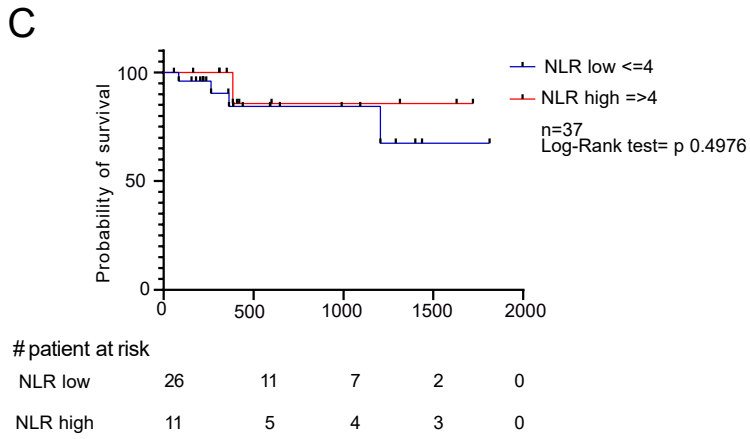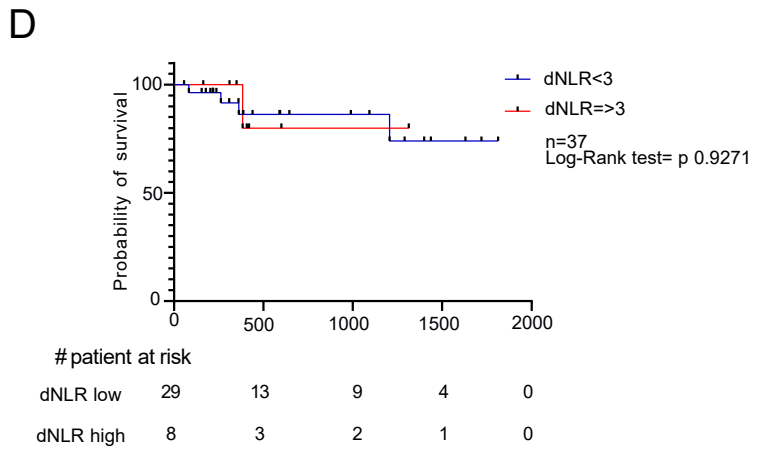

Supplement: Supplementary file 10 — Additional file 10: Supplementary Data 10. Impact of CD8+/FoxP3+ ratio, metastatic lymph node invasion, NLR and dNLR on overall survival. Kaplan-Meier curves displaying the overall survival starting at the date of the surgery according to (A) CD8+/FoxP3+ ratio, (B) metastatic lymph node invasion, (C) NLR and (D) dNLR. NLR: neutrophil-to-lymphocyte ratio; dNLR:derived neutrophil-to-lymphocyte ratio. [file 13046_2023_2897_MOESM10_ESM.pdf]

# Supplementary Data 12

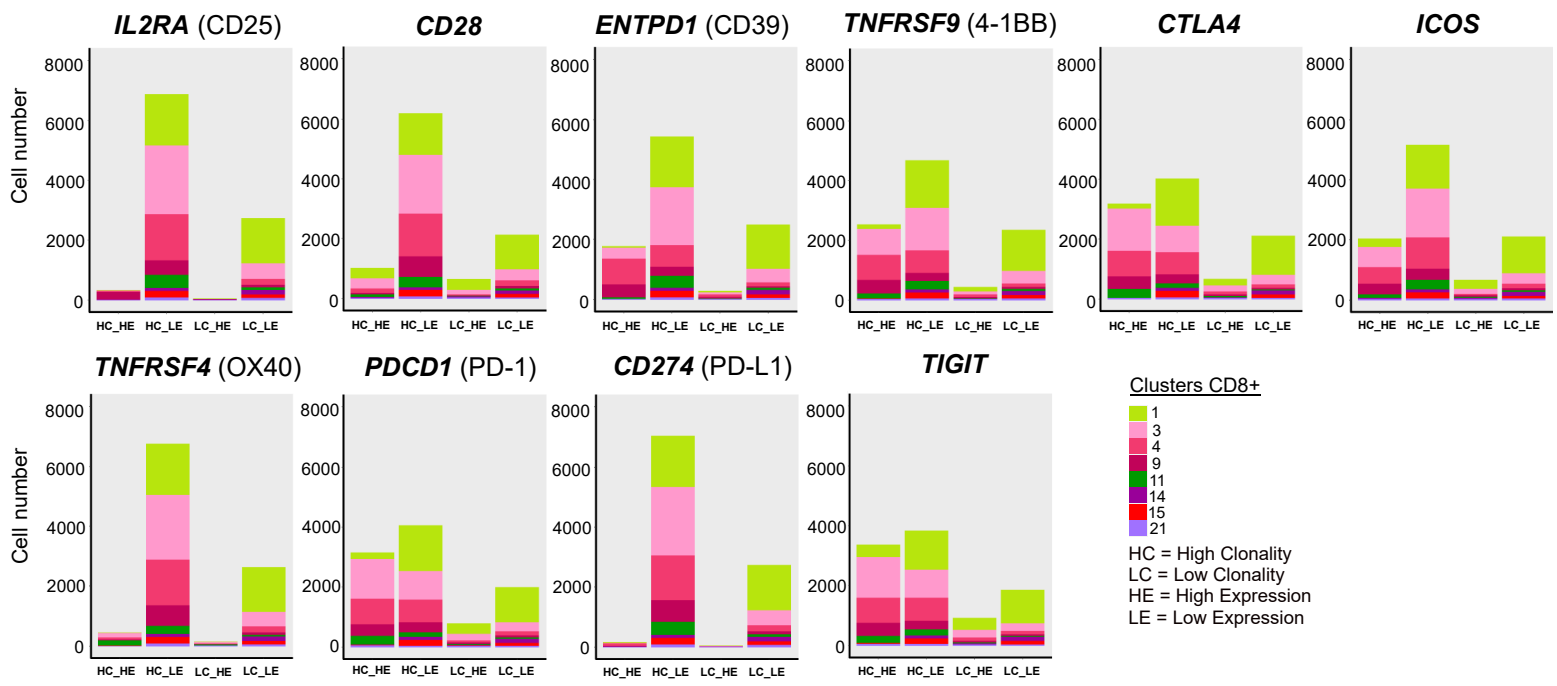

Supplement: Supplementary file 12 — Additional file 12: Supplementary Data 12. Distribution of CD8+ T cells according to ICP expression level and clonality. Stacked bar chart showing the distribution of T cells from CD8+ clusters according to the level of ICP expression (above the median expression level (HM) or below the median expression level (LM)) and the expansion status (LC or HC). Median expression level was calculated independently for each sample and for each ICP. [file 13046_2023_2897_MOESM12_ESM.pdf]
