# Supplementary material for: Immune checkpoints are predominantly co-expressed by clonally expanded CD4+FoxP3+ intratumoral T-cells in primary human cancers
Source: J Exp Clin Cancer Res. 2023 Dec 6;42:333. doi: 10.1186/s13046-023-02897-6 (PMC10699039; doi:10.1186/s13046-023-02897-6)
Supplement: Supplementary file 3 — Additional file 3: Supplementary Data 3. Patient characteristics. [file 13046_2023_2897_MOESM3_ESM.pdf]

Supplementary Data 3

		MM*		NSCLC		RCC#		HNSCC		EOC		UC		HCC		NET		Thyr	
		n	%	n	%	n	%	n	%	n	%	n	%	n	%	n	%	n	%
Total		7	9%	9	12%	12	16%	11	14%	22	29%	12	16%	2	3%	1	1%	1	1%
Gender	Female	3	43%	5	56%	2	17%	4	36%	22	100%	1	8%	0	0%	1	100%	1	100%
	Male	4	57%	4	44%	10	83%	7	64%	0	0%	11	92%	2	100%	0	0%	0	0%
	Missing	0	0%	0	0%	0	0%	0	0%	0	0%	0	0%	0	0%	0	0%	0	0%
Age (years)	Median	48	-	68	-	60	-	63	-	55	-	76	-	75	-	50	-	45	-
	Range	39-63	-	63-79	-	33-76	-	21-89	-	19-74	-	47-89	-	71-78	-	-	-	-	-
	Missing	0	0%	0	0%	0	0%	0	0%	0	0%	0	0%	0	0%	0	0%	0	0%
Metastatic Lymph Nodes	N+	7	100%	5	56%	1	8%	8	73%	11	50%	5	42%	0	0%	1	100%	0	0%
	N-	0	0%	4	44%	11	92%	2	18%	5	23%	5	42%	0	0%	0	0%	1	100%
	Nx	0	0%	0	0%	0	0%	1	9%	6	27%	0	0%	2	100%	0	0%	0	0%
	Missing	0	0%	0	0%	0	0%	0	0%	0	0%	2	17%	0	0%	0	0%	0	0%
Status at surgery	Primary	0	0%	8	89%	6	50%	11	100%	22	100%	12	100%	0	0%	1	100%	1	100%
	Relapsing	7	100%	0	0%	1	8%	0	0%	0	0%	0	0%	2	100%	0	0%	0	0%
	Missing	0	0%	1	11%	5	42%	0	0%	0	0%	0	0%	0	0%	0	0%	0	0%
Prior systemic treatment	Yes	1	14%	1	11%	2	17%	2	18%	10	45%	1	8%	1	50%	0	0%	0	0%
	No	5	71%	7	78%	10	83%	9	82%	12	55%	9	75%	1	50%	1	100%	1	100%
	Missing	1	14%	1	11%	0	0%	0	0%	0	0%	2	17%	0	0%	0	0%	0	0%
NLR	<4	0	0%	3	33%	3	25%	9	82%	15	68%	0	0%	1	50%	1	100%	0	0%
	≥4	0	0%	5	56%	5	42%	1	9%	5	23%	0	0%	0	0%	0	0%	0	0%
	Missing	7	100%	1	11%	4	33%	1	9%	2	9%	12	100%	1	50%	0	0%	1	100%
dNLR	<3	0	0%	6	67%	4	33%	9	82%	17	77%	0	0%	1	50%	1	100%	0	0%
	≥3	0	0%	2	22%	4	33%	1	9%	3	14%	0	0%	0	0%	0	0%	0	0%
	Missing	7	100%	1	11%	4	33%	1	9%	2	9%	12	100%	1	100%	0	0%	1	100%
Assays	Cytometry T cells	7	100%	7	78%	12	100%	10	91%	21	95%	12	100%	1	50%	1	100%	1	100%
	Cytometry ICPs	7	100%	6	67%	7	58%	8	73%	4	18%	0	0%	1	50%	1	100%	1	100%
	scRNAseq	0	0%	2	22%	0	0%	1	9%	1	5%	0	0%	1	50%	0	0%	0	0%

MM: Metastatic Melanoma; NSCLC: Non-Small Cell Lung Cancer; RCC: Renal Cell Carcinoma; HNSCC: Head and Neck Squamous Cell Carcinoma; EOC: Epithelial Ovarian Cancer; UC: Urothelial Carcinoma; HCC Hepatocellular Carcinoma; NET: Neuroendocrine Tumor; Thyr: thyroid cancer; NLR: Neutrophil-to-Lymphocyte Ratio; dNLR: derived Neutrophil-to-Lymphocyte Ratio; * all samples analyzed were metastatic lymph nodes; # 1/12 samples was a metastatic lymph node.
