# Supplementary material for: Immune checkpoints are predominantly co-expressed by clonally expanded CD4+FoxP3+ intratumoral T-cells in primary human cancers
Source: J Exp Clin Cancer Res. 2023 Dec 6;42:333. doi: 10.1186/s13046-023-02897-6 (PMC10699039; doi:10.1186/s13046-023-02897-6)
Supplement: Supplementary file 11 — Additional file 11: Supplementary Data 11. Assignment of single-cell clusters to T-cell lineages according to the expression of canonical gene markers. [file 13046_2023_2897_MOESM11_ESM.pdf]

Supplementary Data 11

	0	2	5	8	1	3	4	9	11	14	15	21	7
	CD4+Effector Memory	$CD4^{+}$ regulatory	CD4+ TFH/TEMRA	CD4+Stem cells like memory	CD8+Effector Memory	CD8+ Exhausted	CD8+Cytotoxic	CD8+ Cytotoxic resident	CD8+ Exhausted TNFRS18/LAG3	CD8+ MAIT	CD8+ Cytotoxic GNYL	CD8+ prolifération	Tγδ/NK
CD3E	+	+	+	+	+	+	+	+	+	+	+	+	+
CD4	+	+	+	+									
CD8A					+	+	+	+	+	+	+	+	+
CD8B					+	+	+	+	+	+	+	+	+
FOXP3		+											
TCF7	-			+									
PRDM1			+										
MAF			+										
STAT4					+								
LEF1	-			+									
GZMA							+	+			+		+
GZMB							+	+			+		+
GZMH							+	+			+		+
GZMK					+		+			+			
PRF1							+	+			+		+
CRTAM							+						
GNLY								+			+		
CXCL13			+			+			+				
CCL5			+										
IFNG						+			+				
PDCD1			+			+		+	+				
CTLA4			+			+		+	+				
TIGIT						+			+				
HAVCR2						+			+				
LAG3						+			+				
TNFRS18									+				
ICOS			+										
KI67												+	
TUBB												+	
STMN1												+	
IL2RA		+						+					
IL2RB	-			+									+
CD28				+									
CD27				+									
FAS				+									
ITGB7								+					
ITGAE								+					
LAYN								+					
KLRC1													+
KLRB1										+			
SELL	-			+	-								
CCR7	-			+	-								
IL7R	+			+	+								
NCR3										+			
TRAV1-2										+			
SLC4A10										+			
FCGR3A													+
TYROBP													+
FCER1G													+
